# Genomic and transcriptomic dynamics in the stepwise progression of lung adenocarcinoma

**DOI:** 10.1038/s41422-025-01200-w

**Published:** 2025-12-04

**Authors:** Fangqiu Fu, Jun Shang, Yueren Yan, He Jiang, Han Han, Hui Yuan, Zhendong Gao, Jingcheng Yang, Jian Gao, Jun Wang, Yunjian Pan, Yicong Lin, Ting Ye, Yiliang Zhang, Yawei Zhang, Jiaqing Xiang, Hong Hu, Zhiwei Cao, Yuanting Zheng, Yuan Li, Yang Zhang, Li Jin, Leming Shi, Haiquan Chen

**Affiliations:** 1 Department of Thoracic Surgery and State Key Laboratory of Genetics and Development of Complex Phenotypes, Fudan University Shanghai Cancer Center, Shanghai, China; 2 Institute of Thoracic Oncology, Fudan University, Shanghai, China; 3 Department of Oncology, Shanghai Medical College, Fudan University, Shanghai, China; 4 State Key Laboratory of Genetic Engineering, School of Life Sciences, Human Phenome Institute and Shanghai Cancer Center, Fudan University, Shanghai, China; 5 International Human Phenome Institutes (Shanghai), Shanghai, China; 6 School of Life Sciences, Fudan University, Shanghai, China; 7 Department of Pathology, Fudan University Shanghai Cancer Center, Shanghai, China

**Keywords:** Cancer genomics, Non-small-cell lung cancer

## Abstract

Lung adenocarcinoma (LUAD) progresses from pre-invasive to invasive stages, as well as from ground-glass opacities (GGOs) to solid nodules. However, the dynamic genomic and transcriptomic changes underlying LUAD progression are incompletely understood. Here, we performed whole-genome and transcriptome sequencing on 1008 LUAD samples from 954 patients who underwent surgery at Fudan University Shanghai Cancer Center, with comprehensive follow-up data. There was one atypical adenomatous hyperplasia, 42 adenocarcinomas in situ, 116 minimally invasive adenocarcinomas, and 849 invasive adenocarcinomas spanning all pathological stages. *EGFR* was the most frequently mutated gene in the study cohort, followed by *TP53, RBM10, KRAS*, and *KMT2D*. Mutation frequencies of tumor suppressor genes, such as *TP53, RB1, MGA, KEAP1*, and *STK11*, increased as the disease progressed to higher stages. A higher level of genomic instability was seen in LUAD compared with AAH/AIS/MIA samples, characterized by a higher tumor mutation burden, increased somatic copy number alteration burden, and increased structural variation burden. Notably, MAP2K1 E102–I103 deletion was frequently observed in pre-invasive samples, which endowed alveolar type II cells with increased growth potential and initiated tumor formation, suggesting that it is a potential driver mutation of LUAD. In summary, our study highlights key molecular changes during the stepwise progression of LUAD, provides insights into the identification of novel therapeutic targets, and helps to define the curative time window for this disease.

## Introduction

Lung cancer is the leading cause of cancer-related death worldwide, presenting a multifaceted challenge in cancer research.^1^ Among the various pathological subtypes, lung adenocarcinoma (LUAD) is the most common, accounting for > 45% of all lung cancer cases, and its incidence is still rising.^2^ From a histological perspective, LUAD is generally thought to progress from atypical adenomatous hyperplasia (AAH) to adenocarcinoma in situ (AIS), minimally invasive adenocarcinoma (MIA), and eventually invasive adenocarcinoma (ranging from stage I to stage IV disease). On CT imaging, some LUADs appear to progress from ground-glass opacities (GGOs) to part-solid nodules containing both GGO and solid components, and finally to solid nodules.^3–5^ Studies have demonstrated an association between radiological appearance and pathological stage: most AIS/MIA lesions tend to present as pure GGOs, whereas the presence of solid components on CT images is correlated with increased histological invasiveness.^6^

Pre-invasive stages of LUAD, such as AIS and MIA, as well as LUADs that present as pure GGOs, have a nearly 100% survival rate following complete surgical resection, leading to the characterization of GGO-like LUADs as an inert subtype due to their slow growth and favorable prognosis.^6–9^ Nonetheless, the 5-year survival rate for invasive adenocarcinoma drops dramatically as the disease develops from stage I to stages II, III, and IV, and from pure GGOs to solid nodules.^10^ With the help of next-generation sequencing, the mutation frequencies of major oncogenes and tumor suppressor genes in LUAD have been characterized.^11–14^ However, most studies have focused on invasive LUAD, with limited research on the key molecular changes that occur during the progression of LUAD. Therefore, it is crucial to uncover the dynamic changes in genomic and transcriptomic profiles that occur as LUAD progresses and to obtain evidence for the optimal curative window for surgical intervention that offers a definitive cure while avoiding overtreatment.^15^

Metastasis is a critical aspect of tumor progression and a leading cause of lung cancer-related mortality.^16,17^ Studies have shown that tumor subclones that harbor certain driver mutations have greater metastatic potential and that metastatic organotropism is associated with specific genetic changes.^12,18–20^ Understanding how the genetic profile of LUAD may influence the pattern of metastatic spread is pivotal for refining treatment, predicting disease outcomes, and planning follow-up strategies, prompting us to examine the intricate landscape of site-specific metastasis in LUAD.

In this study, we performed whole-genome sequencing (WGS) and RNA sequencing (RNA-seq) of 1008 LUAD samples encompassing all pathological and radiological features, together with matched adjacent normal lung tissues, from 954 patients with complete follow-up data. Our objective was to provide an extensive exploration of the dynamically changing genomic and transcriptomic profiles associated with LUAD progression, from inert pre-invasive diseases to invasive stages and finally to late-stage diseases with loco-regional and distant metastasis. Through this comprehensive study, we aim to better define therapeutic windows, inform strategies for disease prevention, and identify potential targets for drug development.

## Results

### Clinical and pathological characteristics of patients

**Fig. 1:**
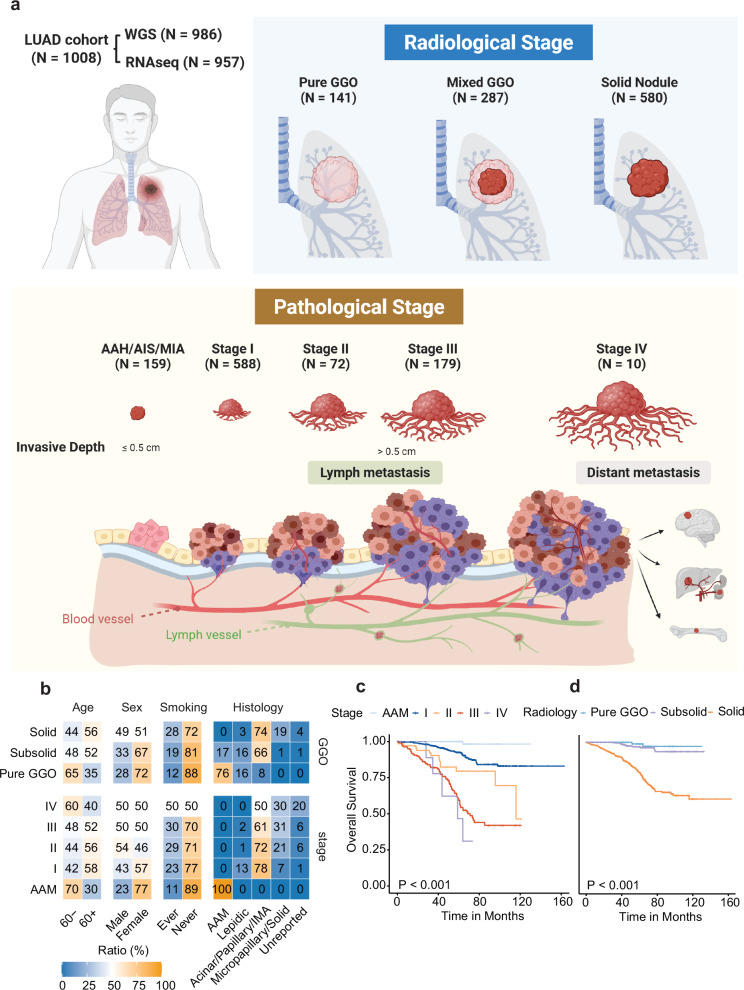
Study design and clinicopathological characteristics of patients in the study cohort. **a** 1008 tumors from 954 patients who underwent surgery between August 2011 and March 2019 at the Department of Thoracic Surgery, Fudan University Shanghai Cancer Center, were retrospectively included in this study. WGS and RNA-seq were performed on all tumors and their paired adjacent normal lung tissues. After quality control, 986 samples with WGS data and 968 samples with RNA-seq data were deemed satisfactory for downstream analyses. The numbers of samples from different radiological and pathological stages were recorded. **b** Proportions of patients of different ages, sexes, smoking histories, and histologies stratified by radiological and pathological stages. **c** Kaplan–Meier curves showing overall survival (OS) of patients with different pathological stages. **d** Kaplan–Meier curves showing OS of patients with different radiological appearances. The log-rank test was used to calculate the statistical significance of differences.

A total of 1008 tumors from 954 patients were included in this study. The median recurrence-free survival (RFS) was 56.0 months, and the median overall survival (OS) was 60.8 months. Among the patients, 548 (57.4%) were female and 406 (42.6%) were male. In addition, 731 patients (76.6%) were self-reported never-smokers. The median age was 60 years (range: 23–89). Of all the lesions, 141 (14.0%) appeared as pure GGOs, 287 (28.5%) appeared as mixed GGOs, and 580 (57.5%) appeared as solid nodules on CT scans. The pathological classifications included one case of atypical adenomatous hyperplasia (AAH, 0.1%), 42 cases of adenocarcinoma in situ (AIS, 4.2%), 116 cases of minimally invasive adenocarcinoma (MIA, 11.5%), and 849 cases of LUAD (84.2%, Fig. 1a).

Age, sex, smoking status, and predominant subtypes of LUAD were recorded and compared. Notably, there were more female patients with early-stage LUAD, and the proportion of patients with a smoking history increased as the disease progressed to higher stages (Fig. 1b). Subtypes considered high-grade and associated with poor prognosis, such as the micropapillary and solid subtypes,^21^ were more commonly seen in the later stages of the disease (Fig. 1b). Pre-invasive lesions were associated with perfect survival after complete surgical resection, as evidenced by excellent survival in patients with AAH/AIS/MIA (Fig. 1c). Furthermore, OS was significantly higher in patients with GGO components on CT scans than in those with purely solid nodules (Fig. 1d).

### Landscape of somatic mutations in LUAD at different pathological stages

**Fig. 2:**
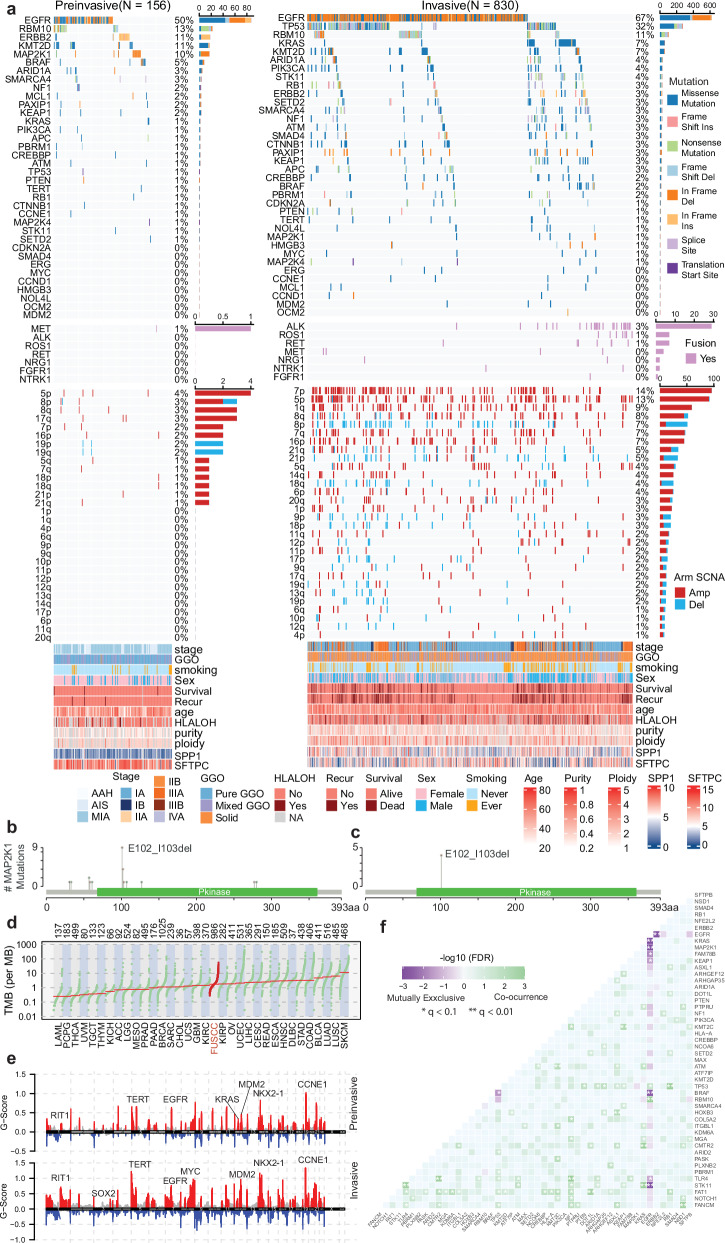
Genomic changes during the progression of LUAD. **a** Mutation landscape of pre-invasive (AAH/AIS/MIA, left) and invasive adenocarcinoma (right) samples. **b** *MAP2K1* mutations identified in the study cohort. **c** *MAP2K1* mutations identified in our previous study of 98 pre-invasive and 99 invasive adenocarcinoma samples. **d** Comparison of tumor mutation burden between samples in this cohort and TCGA cohorts. **e** Landscape of somatic copy number alterations (SCNAs) in this study cohort, with pre-invasive samples at the top and invasive samples at the bottom. **f** Co-mutational status, showing the mutual exclusivity and co-occurrence of major oncogenes and tumor suppressor genes. Statistical significance was assessed using Fisher’s exact test.

We identified somatic mutations of major driver genes and tumor suppressor genes in our study cohort. We first divided the samples into pre-invasive (AAH/AIS/MIA) and invasive (LUAD) groups and found that the most frequently mutated gene in pre-invasive samples was *EGFR* (50%), followed by *RBM10* (13%), *ERBB2* (11%), *KMT2D* (11%), *MAP2K1* (10%), and *BRAF* (5%). By contrast, the most frequently mutated gene in LUAD samples was *EGFR* (67%), followed by *TP53* (32%), *RBM10* (11%), *KRAS* (7%), *KMT2D* (7%), and *ARID1A* (4%, Fig. 2a).

Available ClinVar pathogenicity information and functional impact predictions obtained with SIFT and PolyPhen-2 were also examined (Supplementary information, Fig. S1a–c). Interestingly, the frequencies of EGFR L858R and exon 19 deletion mutations and KRAS G12C mutation were higher in invasive samples than in pre-invasive samples, but there was no significant difference in the frequency of KRAS G12D (Supplementary information, Fig. S1d). Notably, we identified a mutation hotspot in *MAP2K1* with a median variant allele frequency of 0.15 (Supplementary information, Fig. S1e), a potential candidate driver mutation of LUAD, which was previously reported as a missense mutation in 2 of 230 (0.9%) patients with invasive LUAD.^11^ In our study, 13 cases with MAP2K1 p.E102–I103 deletion were identified, 11 of which were AIS/MIA samples (Fig. 2a, b). This mutation hotspot was also identified in our previous study, which used whole-exome sequencing (WES) to decipher the genomic profiles of pre-invasive and invasive LUAD.^22^ In that study, 4 of 197 (2.0%) LUAD patients harbored MAP2K1 p.E102–I103 deletion, and all 4 of these samples were pre-invasive (Fig. 2c). Because it was mutually exclusive with known driver mutations of LUAD, such as EGFR, KRAS, and BRAF, MAP2K1 p.E102-I103 deletion might have driving potential in LUAD.

The median tumor mutation burden (TMB) across the entire study was 1.73 mutations per megabase (Mb), with 1.20/Mb for AAH/AIS/MIA and 1.81/Mb for LUAD (Fig. 2d; Supplementary information, Fig. S2a). TMB increased as tumors progressed to higher radiological and pathological stages (Supplementary information, Fig. S2b, c).

### MAP2K1 p.E102–I103 deletion might drive lung adenocarcinoma formation in alveolar type II (ATII) organoids

**Fig. 3:**
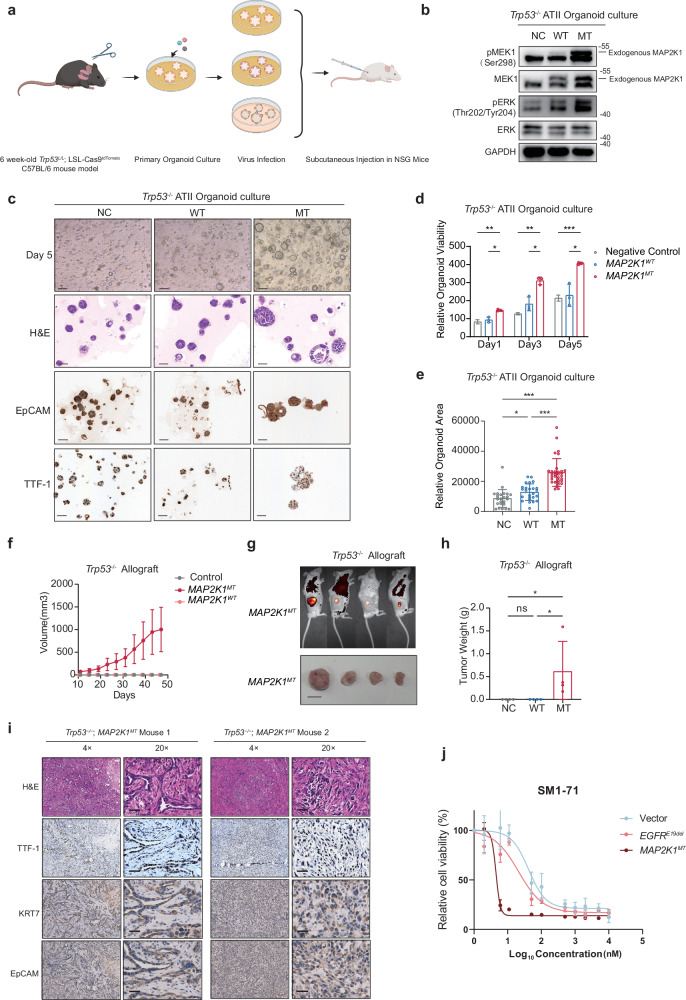
In vitro and in vivo experiments to test the oncogenicity of *MAP2K1*^*ΔE102−I103*^. **a** Study design of in vitro and in vivo experiments. **b** Western blot analysis of phosphorylated and total Mek1 and Erk in *Trp53*^−/−^–ATII organoids. NC negative control group, WT group overexpressing WT *MAP2K1*, MT group overexpressing *MAP2K1*^*ΔE102−I103*^. **c** Morphological changes (top), H&E staining (second row), and IHC of EpCAM (third row) and TTF-1 (bottom) in *Trp53*^−/−^–ATII organoids after 5 days of culture. Scale bar, 50 µm. **d** Relative viability of organoids on days 1, 3, and 5. *n* = 3 biological replicates. **e** Relative organoid area of organoids on day 5. **f**–**h** Growth curve (**f**), end point illustration (**g**, scale bar, 1 cm), and tumor weight (**h**) of *Trp53*^−/−^ (*n* = 4), *Trp53*^−/−^;*MAP2K1*^*WT*^ (*n* = 4), and *Trp53*^−/−^;*MAP2K1*^*MT*^ (*n* = 4) allografts. **i** H&E staining and IHC of TTF-1, KRT7, and EpCAM of the *Trp53*^−/−^;*MAP2K1*^*MT*^ organoid allograft at the experiment endpoint (day 48). Scale bar, 50 µm. **j** Efficacy of SM1-71, an inhibitor of MAP2K1, in treating *MAP2K1*^*MT*^, *MAP2K1*^*WT*^, and negative-control Ba/F3 cells. *p < 0.05, **p < 0.01, ***p < 0.001.

Given the oncogenic potential of *MAP2K1* and the fact that its hotspot mutation p.E102–I103 deletion was mutually exclusive with other oncogenic mutations (Fig. 2f) and significantly enriched in pre-invasive LUAD (Fig. 2a), we performed in vitro and in vivo experiments to assess its oncogenicity. Since LUAD is widely believed to originate ATII cells, we generated mouse lung ATII organoid cultures from C57BL/6J *Trp53*^*L/L*^;*LSL-Cas9*^*tdTomato*^ mice, in which the floxed *Trp53* gene could be conditionally inactivated and tdTomato-Cas9 could be conditionally activated by Cre recombinase. These *Trp53*^*L/L*^;*Cas9* ATII organoids formed spheres after 5 days of culture. Following expansion, the organoids were infected with adenovirus-Cre (Ad-Cre). The tdTomato^+^ cells were then sorted by flow cytometry, generating *Trp53*^*–/–*^ ATII organoids (Fig. 3a).

To investigate the oncogenic potential of the *MAP2K1*^*ΔE102−I103*^ mutation, we overexpressed the *MAP2K1*^*ΔE102−I103*^ mutant (MT) and wild-type (WT) *MAP2K1* in the *Trp53*^*–/–*^ organoids, alongside a negative control lentivirus (Fig. 3a). The mutant and WT *MAP2K1* cDNA sequences were verified by genomic sequencing (Supplementary information, Fig. S3). Western blot analysis showed that overexpression of *MAP2K1*^*ΔE102−I103*^ significantly upregulated phosphorylated MEK1 (pMEK1) and phosphorylated ERK (pERK) — the direct downstream molecule of MEK in the MAPK signaling pathway (Fig. 3b). When we cultured the organoids in vitro for 5 days (Fig. 3c) and performed viability assays, we found that the proliferation capability of the *MAP2K1*^*ΔE102−I103*^ group was significantly higher than that of the WT and NC groups (Fig. 3d). Morphologically, the spheroids that formed in the *MAP2K1*^*ΔE102−I103*^ group were also significantly larger than those of the other two groups (Fig. 3e). All three groups were strongly positive for TTF-1 and EpCAM in immunohistochemistry (IHC) assays (Fig. 3c). These data demonstrated that the *MAP2K1* mutation could promote proliferation and adeno-differentiation of ATII organoids in vitro.

To assess the tumorigenic capability of genetically engineered organoids in vivo, we implanted the *Trp53*^−/−^;*MAP2K1*^*MT*^ organoids, *Trp53*^−/−^;*MAP2K1*^*WT*^ organoids, and control *Trp53*^−/−^ organoids into the lower flanks of NOD/ShiLtGpt-Prkdc^em26cd52^Il2rg^em26cd22^/cpt (NSG) mice (Fig. 3a). Strikingly, 10 days after injection, tumors formed in mice injected with *Trp53*^−/−^;*MAP2K1*^*MT*^ organoids but not in those injected with the *Trp53*^−/−^ organoids or *Trp53*^−/−^;*MAP2K1*^*WT*^ organoids (Fig. 3f–h). Importantly, this suggests that loss of *Trp53* alone or combined with overexpression of *MAP2K1*^*WT*^ in ATII organoids is not sufficient to generate LUAD in vivo. Histologic analysis of *Trp53*^−/−^;*MAP2K1*^*MT*^ tumors revealed strong expression of TTF-1 and EpCAM, consistent with LUAD markers (Fig. 3i). Furthermore, SM1-71, an inhibitor of MAP2K1, showed better efficacy in treating *MAP2K1*^*MT*^ Ba/F3 cells than *EGFR*^*E19de*^ or negative-control Ba/F3 cells (Fig. 3j). In summary, these findings provide evidence that *MAP2K1*^*ΔE102−I103*^, in the absence of *Trp53*, can drive LUAD formation in vivo and is targetable in cancer treatment.

We next compared the selective advantage conferred by *MAP2K1*^*ΔE102−I103*^ vs two major driver mutations in LUAD, *EGFR*^*ΔE746−A750*^ and *KRAS*^*G12D*^ (Supplementary information, Fig. S4a). When mCherry-labeled Ba/F3 cells harboring *MAP2K1*^*ΔE102−I103*^ were co-cultured at a 1:1 ratio with GFP-labeled Ba/F3 cells carrying either *EGFR*^*ΔE746−A750*^ or *KRAS*^*G12D*^, we observed a significant decline in the proportion of cells with *MAP2K1*^*ΔE102−I103*^ by day 3, with near-complete dominance of *EGFR*^*ΔE746−A750*^ or *KRAS*^*G12D*^ clones by day 7 (Supplementary information, Fig. S4b). Further western blot analysis revealed similar pERK levels in *MAP2K1*^*ΔE102−I103*^ and *EGFR*^*ΔE746−A750*^ cells, and pAKT levels were comparable between *MAP2K1*^*ΔE102−I103*^ and vector control cells (Supplementary information, Fig. S4c). These results indicated that the MEK/ERK pathway was activated in *MAP2K1*^*ΔE102−I103*^ Ba/F3 cells, whereas the PI3K/AKT pathway was not. Taken together, these findings suggest that *EGFR*^*ΔE746−A750*^ and *KRAS*^*G12D*^ provide a stronger selective advantage over *MAP2K1*^*ΔE102−I103*^, which may explain why *MAP2K1*^*ΔE102−I103*^ is predominantly enriched in early-stage LUAD.

### Comparison of genomic features of LUAD at different developmental stages

**Fig. 4:**
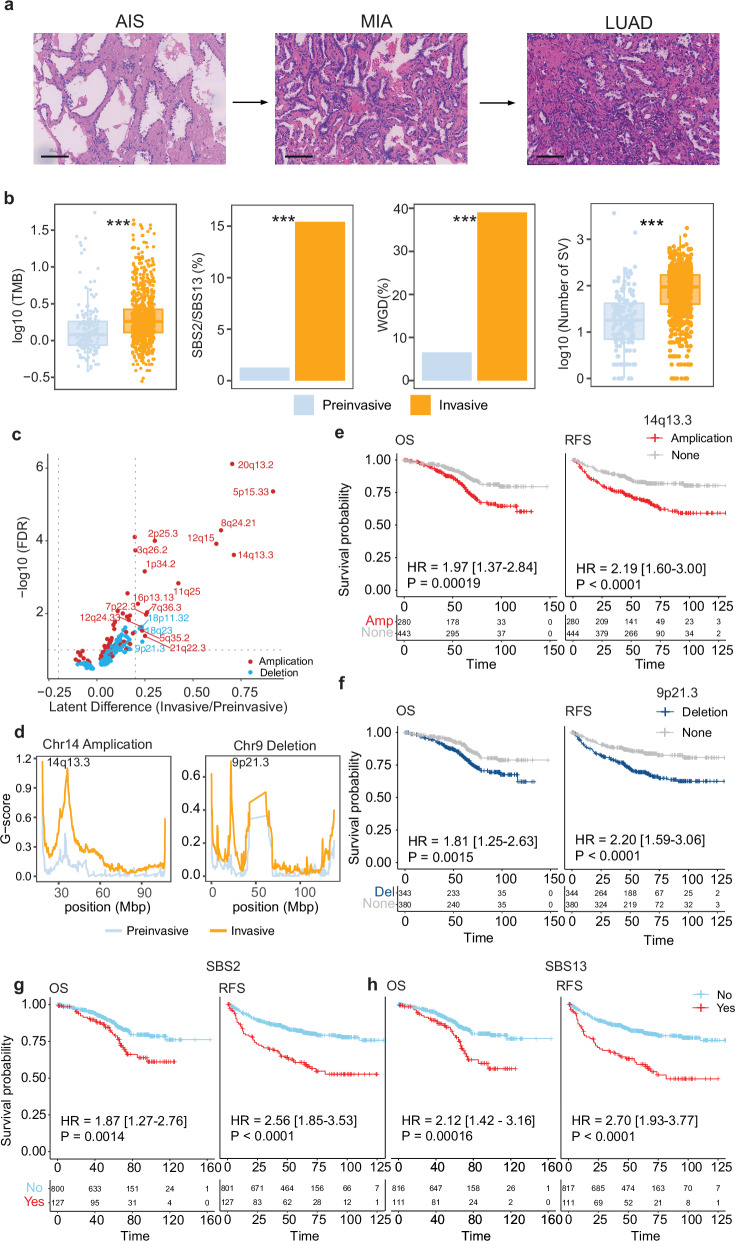
Comparison of genomic features of LUADs at different developmental stages. **a** H&E-stained images of AIS, MIA, and LUAD. Scale bar, 100 µm. **b** Left to right: comparisons of tumor mutation burden (TMB), APOBEC signature (SBS2/13) activities, whole-genome doubling (WGD) events, and structural variations (SVs) between pre-invasive and invasive adenocarcinoma. **c** Comparison of SCNA events between pre-invasive and invasive adenocarcinoma. **d** Comparison of G scores of 14q13.3 gain (left) and 9p21.3 loss (right) between pre-invasive and invasive adenocarcinoma. **e** Kaplan–Meier curves showing OS (left) and RFS (right) of patients with chromosome 14q13.3 amplifications. **f** Kaplan–Meier curves showing OS (left) and RFS (right) of patients with chromosome 9p21.3 losses. **g** Kaplan–Meier curves showing OS (left) and RFS (right) of patients with different SBS2 signature activities. **h** Kaplan–Meier curves showing OS (left) and RFS (right) of patients with different SBS13 signature activities. Statistical significance was assessed using the Wilcoxon test, Fisher’s exact test, and the log-rank test. ***p < 0.001.

Before developing into LUAD, cells are generally thought to progress through the stages of AAH, AIS, and MIA (Fig. 4a). Somatic copy number alterations (SCNAs) were identified and categorized as gains or losses in pre-invasive and invasive samples. In pre-invasive samples, the most frequently observed SCNA event was a gain in chromosome 5p, followed by gains in chromosomes 8p, 8q, 17q, 7p, and 16p (Fig. 2a, e). Copy number losses in chromosomes 19p and 19q were also identified in pre-invasive samples (Fig. 2a, e). In invasive samples, the most frequent gains were observed in chromosomes 7p, 5p, 1q, 8q, and 7q, and the most frequent losses were in chromosomes 8p and 21p (Fig. 2a, e).

We observed a higher level of genomic instability in LUAD than in AAH/AIS/MIA, characterized by an increased TMB, a higher degree of arm-level SCNAs, and an increased level of structural variations (SVs) as tumors progressed to more advanced stages (Fig. 4b). Notably, a sharp increase in SCNA burden was seen between AAH/AIS/MIA samples and stage I LUAD samples when LUADs were divided by their pathological stage (Supplementary information, Fig. S5a). Whole-genome doubling (WGD) events were also more frequent in LUAD than in AAH/AIS/MIA (Fig. 4b). Moreover, a more pronounced increase in the number of SVs was observed between AAH/AIS/MIA samples and stage I LUAD samples (Fig. 4b; Supplementary information, Fig. S5b). We performed an analysis of SVs and further examined their association with LUAD progression. It was evident that abnormal SVs, including different lengths and types of SVs, breakage-fusion-bridge (BFB) structural variants, and chromothripsis events, progressively increased throughout the pathological and radiological progression (Supplementary information, Fig. S6). This indicates that the progression of LUAD is accompanied by an accumulation of abnormal SV events.

To gain a deeper understanding of the progression and prognostic value of SCNA events, we compared the frequency of focal copy number alterations between AAH/AIS/MIA and LUAD samples (Fig. 4c). A univariate Cox regression model revealed that gains of 14q13.3, 3q26.2, 7p22.3, 7q36.3, and 11q25 and loss of 9p21.3 were associated with worse RFS and OS (Supplementary information, Fig. S7). In a multivariate Cox regression model adjusted for age, sex, smoking status, and stage, these six focal copy number alterations remained independent prognostic factors for RFS, and gain of 14q13.3 and 3q26.2 and loss of 9p21.3 remained independent prognostic factors for OS (Supplementary information, Fig. S8). Among the 18 significantly different focal SCNA events, amplification of 14q13.3, where *NKX2-1* is located, and loss of 9p21.3, where *CDKN2A/2B* are located, had the most significantly different frequencies between AAH/AIS/MIA and LUAD samples (Fig. 4d). Survival analyses indicated that patients harboring 14q13.3 amplifications or 9p21.3 losses had significantly worse RFS and OS than those without (Fig. 4e, f).

Therefore, deciphering and comparing the genomic features at each of these stages is crucial for understanding the molecular changes that take place during disease progression. We next compared the genomic and transcriptomic alterations associated with LUAD at different developmental stages. *EGFR*, *KRAS*, and *ALK* had higher mutation frequencies as the disease progressed from pre-invasive (AAH/AIS/MIA) to invasive stages (Supplementary information, Fig. S9a). Mutations in genes of the RTK-RAS pathway were also more common in invasive stages (Supplementary information, Fig. S9a). However, the mutation frequency of *ERBB2* and *MAP2K1* was significantly lower in invasive adenocarcinoma compared with pre-invasive lesions (Supplementary information, Fig. S9b). The frequency of mutations in tumor suppressor genes tended to increase as tumor stage progressed, including mutations in *TP53*, *RB1*, *MGA*, *KEAP1*, and *STK11* (Supplementary information, Fig. S9c). Interestingly, the frequency of *KEAP1* mutation remained relatively low until the disease reached stage IV LUAD (Supplementary information, Fig. S9c), suggesting that it is a late event in tumor evolution.^23^ Given that some tumor suppressor genes had low mutation frequencies, we analyzed a combination of all tumor suppressor genes and found a significant increase in mutation frequencies as the disease progressed to higher stages (Supplementary information, Fig. S9c). Human leukocyte antigen loss of heterozygosity (HLA LOH) is a process in which cancer cells shed a portion of their HLA genes, making them less visible to the immune system.^24^ The HLA LOH ratio was low at the pre-invasive stage and began to increase when the disease progressed to the invasive stage (Supplementary information, Fig. S9d).

Mutation signatures reflect normal cell biology, environmental exposures, and neoplastic progression. They are associated with survival outcomes and have therapeutic implications for patients with various cancer types.^25–29^ In this study cohort, signatures of single-base substitutions (SBS) were identified and compared among groups. We found that the activity of the APOBEC signature (SBS2/13), SBS4, and SBS16 tended to increase as the disease progressed to higher stages (Fig. 4b; Supplementary information, Fig. S10a, b). In addition, patients with higher APOBEC signature activity, as well as elevated SBS4 and SBS16 activities, had significantly worse RFS and OS, and this significant difference in survival was also observed in a multivariate Cox regression model with sex, age, and smoking status as confounding factors (Fig. 4g, h; Supplementary information, Fig. S10c).

### Co-mutations and their prognostic value

Driver events in tumors may be mutually exclusive or may tend to co-occur, and their co-mutation status has been reported to have prognostic value.^30,31^ In this large single-institution cohort, we assessed the co-mutation status of major driver genes in LUAD. We found that some known oncogenic mutations in LUAD, such as mutations in *KRAS*, *EGFR*, *ERBB2*, and *BRAF*, were mutually exclusive in our cohort (Fig. 2f). Notably, *MAP2K1* mutations were also mutually exclusive with these major oncogenic mutations (Fig. 2f). Interestingly, mutual exclusivity was observed in certain oncogene–tumor suppressor gene pairs as well. For example, *STK11* and *KEAP1* were mutually exclusive with *EGFR* mutations, and *TP53* was mutually exclusive with *BRAF* mutations (Fig. 2f).

Certain mutations also tended to co-occur, forming specific mutation pairs. In total, 66 co-mutation pairs involving major oncogenes and tumor suppressor genes were identified. These included pairs consisting of one oncogene and one tumor suppressor gene, such as *EGFR* co-mutated with *TP53/RBM10*, and pairs consisting of two tumor suppressor genes, such as *TP53* co-mutated with *KEAP1/ARID1A/RB1/FAT1* (Fig. 2f). Among the co-mutation pairs that contained one oncogene and one tumor suppressor gene with a mutation frequency greater than 3% in the invasive group, 24 pairs were associated with worse RFS and 24 were associated with worse OS (Supplementary information, Fig. S11a, b). For pairs that consisted of two tumor suppressor genes with a mutation frequency greater than 3% in the invasive group, 27 pairs were associated with worse RFS, and 24 were associated with worse OS (Supplementary information, Fig. S11a, b).

Interestingly, co-mutation with *TP53* was the most common factor associated with worse survival outcomes, affecting both RFS and OS in patients (5 oncogenes and 8 tumor suppressor genes for RFS; 5 oncogenes and 8 tumor suppressor genes for OS; Supplementary information, Fig. S11c, d). This was followed by *STK11*, which was also associated with poorer survival outcomes in several co-mutation pairs (3 oncogenes and 5 tumor suppressor genes for RFS; 3 oncogenes and 5 tumor suppressor genes for OS; Supplementary information, Fig. S11c, d). In summary, co-mutation pairs consisting of 1 oncogene and 1 tumor suppressor gene or two tumor suppressor genes hold significant clinical importance and prognostic value in LUAD.

### Comparison of genomic and transcriptomic profiles for LUAD at different radiological stages

**Fig. 5:**
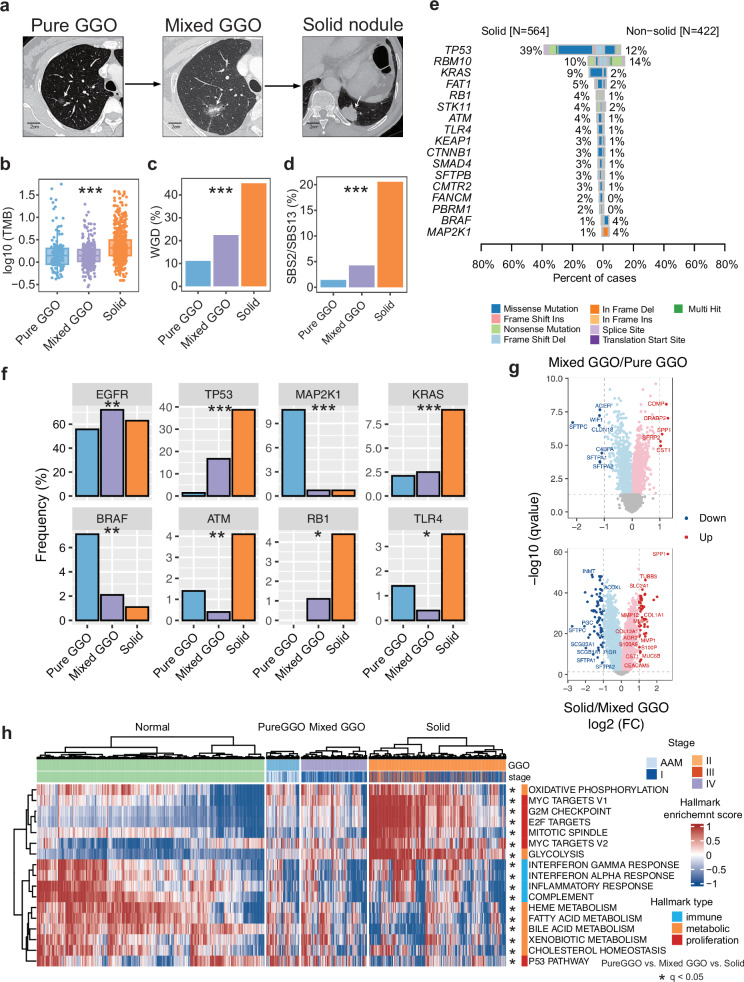
Comparison of genomic and transcriptomic profiles of LUAD at different radiological stages. **a** Radiological appearance of lung adenocarcinomas at different radiological stages. Scale bar, 2 cm. **b** Comparison of TMB among different radiological groups. **c** Comparison of WGD events among different radiological groups. **d** Comparison of SBS2/13 activities among different radiological groups. **e** Comparison of mutation frequencies in driver genes between solid and non-solid samples. **f** Comparison of mutation frequencies in major oncogenes and tumor suppressor genes stratified by different radiological appearances. **g** DEGs between mixed and pure GGOs (top) and between solid nodules and mixed GGOs (bottom). **h** Expression of different pathways in samples with different radiological appearances. Statistical significance was assessed using the Wilcoxon test and Fisher’s exact test. *p < 0.05, **p < 0.01, ***p < 0.001.

Pulmonary tumors may present as pure GGOs, subsolid nodules, or solid nodules on CT images, and radiological findings are correlated with pathological stages and patient survival outcomes (Fig. 5a). To identify key molecular events in the progression of LUAD, we compared genomic and transcriptomic changes among different radiological groups. TMB, WGD, and APOBEC signature activity all increased as the solid components on the CT scans increased (Fig. 5b–d). Compared with non-solid nodules, solid nodules had higher frequencies of mutations in *TP53* (39% vs 12%), *KRAS* (9% vs 2%), *FAT1* (5% vs 2%), *RB1* (4% vs 1%), and *STK11* (4% vs 2%), most of which were tumor suppressor genes. By contrast, solid nodules had lower frequencies of mutations in *RBM10* (10% vs 14%), *BRAF* (1% vs 4%), and *MAP2K1* (1% vs 4%, Fig. 5e). Specifically, *KRAS* mutations were more frequent in solid nodules, whereas *BRAF* mutations were more enriched in pure GGOs (Fig. 5f). Interestingly, *EGFR* mutation frequency did not follow a consistent pattern, with mixed GGOs showing the highest frequency (Fig. 5f). The mutation frequencies of individual tumor suppressor genes, such as *TP53* and *RB1*, followed a monotonically increasing pattern as solid components increased on CT scans (Fig. 5f). The ratio of HLA LOH increased as the tumors progressed into solid nodules (Supplementary information, Fig. S9d).

When identifying differentially expressed genes (DEGs) between mixed and pure GGOs using a 2-fold change threshold and a false discovery rate (FDR) of 0.05, we found 5 significantly upregulated and 6 significantly downregulated genes. When comparing the solid nodules to mixed GGOs, we found 43 significantly upregulated and 67 significantly downregulated genes (Fig. 5g). As tumors progressed, the expression of some pathways followed a stepwise pattern, including those associated with proliferation, immune response, and metabolism (Fig. 5h).

### Key molecular events associated with LUAD progression were identified by integrating pathological, radiological, and genomic alterations

**Fig. 6:**
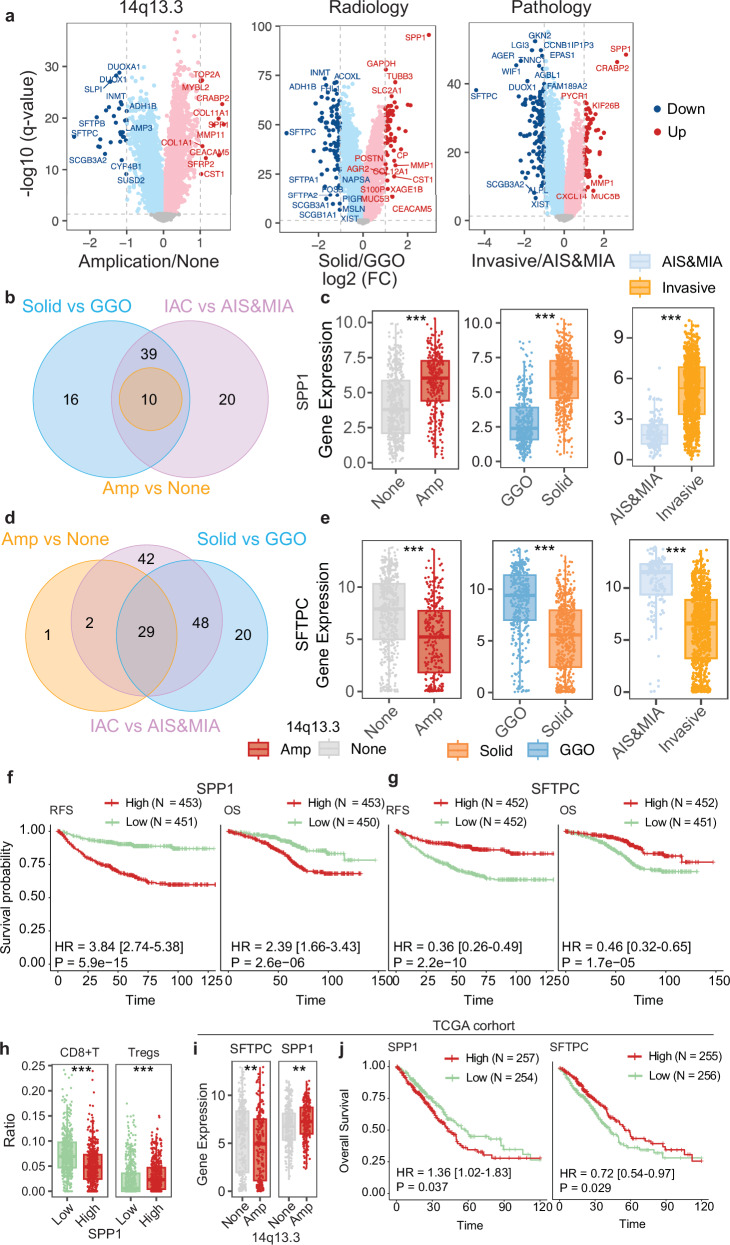
Key transcriptomic changes associated with LUAD progression. **a** DEGs between samples with or without chromosome 14q13.3 amplifications (left), samples manifesting as solid nodules vs GGOs (middle), and invasive vs pre-invasive samples (right). **b** Venn diagram showing significantly upregulated genes in all 3 comparisons. **c** Comparison of *SPP1* expression in samples with or without chromosome 14q13.3 amplifications (left), samples manifesting as solid nodules vs GGOs (middle), and invasive vs pre-invasive samples (right). **d** Venn diagram showing significantly downregulated genes in all 3 comparisons. **e** Comparison of *SFTPC* expression in samples with or without chromosome 14q13.3 amplifications (left), samples manifesting as solid nodules vs GGOs (middle), and invasive vs pre-invasive samples (right). **f** Kaplan–Meier curves showing the OS (left) and RFS (right) of patients with high or low expression of *SPP1*. **g** Kaplan–Meier curves showing the OS (left) and RFS (right) of patients with high or low expression of *SFTPC*. **h** Comparison of CD8^+^ T-cell and Treg infiltration between samples with high and low *SPP1* expression. **i** Comparison of *SFTPC* and *SPP1* expression between samples with or without chromosome 14q13.3 amplification in the TCGA-LUAD cohort. **j** Kaplan–Meier curves showing the OS of patients with high or low expression of *SPP1* (left) and *SFTPC* (right) in the TCGA-LUAD cohort. Statistical significance was assessed using Fisher’s exact test and the log-rank test. **p < 0.01, ***p < 0.001.

We next used transcriptomic data to explore the key molecular events that drive the progression of LUAD and have a prognostic impact. DEGs were identified between AAH/AIS/MIA samples and LUAD samples. Compared with AAH/AIS/MIA samples, 69 genes were upregulated and 121 were downregulated in LUAD samples (Fig. 6a). Similarly, we identified DEGs between solid and GGO samples and found 64 upregulated and 96 downregulated genes in the solid samples (Fig. 6a). As amplification of chromosome 14q13.3 had a significant effect on patient survival (Fig. 4e), we also identified DEGs between samples with and without the amplification of chromosome 14q13.3. There were 6 upregulated and 30 downregulated genes in samples with 14q13.3 amplification compared with those without (Fig. 6a). We found 10 upregulated and 29 downregulated genes that were significantly different in all three comparisons (Fig. 6b–e). Among these, we found that upregulation of *SPP1*, which encodes a secreted protein and cytokine, and downregulation of *SFTPC*, which encodes the pulmonary-associated surfactant protein C, were associated with worse RFS and OS (Fig. 6f, g).

Interestingly, when performing the differential expression analysis in a stepwise manner, we found that *SPP1* upregulation and *SFTPC* downregulation were both significant when comparing stage I LUAD vs AAH/AIS/MIA and stage II vs stage I LUAD (Supplementary information, Fig. S12a). No significant DEGs were identified between stage III vs stage II or stage IV vs stage III, perhaps owing to the smaller sample sizes and similar biological behaviors of tumors at these stages (Supplementary information, Fig. S12a). To confirm our findings, we used genomic and transcriptomic data from the TCGA-LUAD cohort. We found that samples harboring the 14q13.3 amplification had significantly higher expression of *SPP1* and lower expression of *SFTPC*, and that both upregulation of *SPP1* and downregulation of *SFTPC* were associated with worse OS (Fig. 6i, j).

To understand the immune microenvironment of tumors, we used CIBERSORTx, a deconvolution algorithm, to infer the components of the tumor microenvironment using expression signatures of specific cells.^32^ We found that tumors with higher expression of *SPP1* had a more inhibitory immune microenvironment, characterized by fewer infiltrating CD8^+^ cytotoxic cells and more regulatory T cells (Tregs, Fig. 6h). In addition, when comparing the expression of common immune checkpoints, we found higher expression of *HAVCR2* and *LAG3* in tumors with higher *SPP1* expression (Supplementary information, Fig. S12b).

Smoking is a key factor in the oncogenesis of LUAD and has significant prognostic value. We next divided the study cohort into ever-smokers and never-smokers. The most frequently mutated genes in ever-smokers were *EGFR* (54%), *TP53* (40%), *KRAS* (11%), and *RBM10* (9%), and the most frequently mutated genes in never-smokers were *EGFR* (68%), *TP53* (23%), *RBM10* (12%), *KMT2D* (8%), and *KRAS* (5%, Supplementary information, Fig. S13). In both groups, TMB, APOBEC signature, and WGD events were significantly higher (Supplementary information, Fig. S14a–c). For ever-smokers, the mutation frequency of *TP53* was significantly higher in invasive than pre-invasive LUADs (Supplementary information, Fig. S14d). In never-smokers, the mutation frequencies of *EGFR*, *KRAS*, and *TP53* were significantly higher in invasive than pre-invasive LUADs, whereas the mutation frequencies of *ERBB2* and *MAP2K1* were significantly lower in invasive than pre-invasive LUADs (Supplementary information, Fig. S14e). Similar to our findings for the entire cohort, *SPP1* was significantly upregulated in invasive samples compared with pre-invasive samples in both ever- and never-smokers, whereas *SFTPC* was significantly downregulated in invasive samples (Supplementary information, Fig. S14f). In terms of immune cell infiltration, the Treg score was higher in invasive samples from both ever- and never-smokers, whereas the CD8^+^ T score was higher in invasive samples from never-smokers (Supplementary information, Fig. S14g). Amplification of 14q13.3 and deletion of 9p21.3 were more prevalent in LUAD than in pre-invasive lesions, regardless of smoking status (Supplementary information, Fig. S14h).

### LUAD patients with site-specific recurrences had unique genomic and transcriptomic profiles

**Fig. 7:**
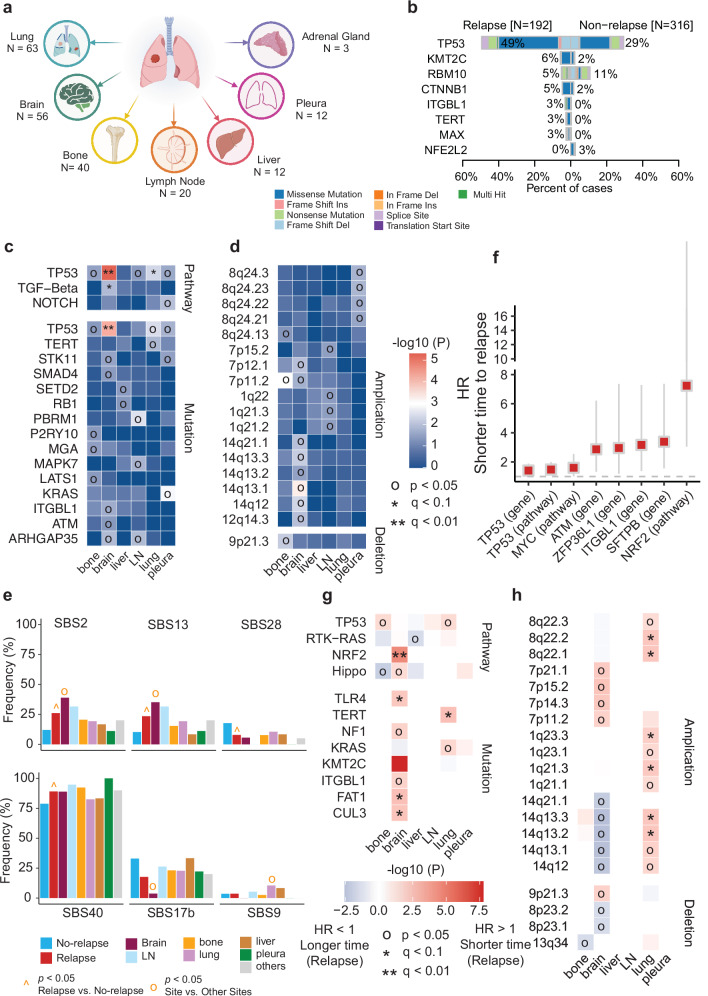
Genomic alterations associated with site-specific metastasis. **a** Overview of patients with specific metastatic sites. **b** Driver mutations with significantly different frequencies between patients with and without disease relapse. **c** Mutations in specific genes and pathways associated with site-specific metastasis. **d** SCNAs associated with specific metastatic sites. **e** Mutation signatures associated with disease relapse and site-specific metastasis. **f** Mutations in specific genes and pathways associated with a shorter time to relapse. **g** Mutations in specific genes and pathways associated with a shorter time to site-specific metastasis. **h** SCNAs associated with a shorter time to site-specific metastasis. Statistical significance was assessed using the Wilcoxon test.

Metastasis is a key hallmark of tumor progression. Lung cancer, harboring distinct genomic alterations, might preferentially metastasize to specific organs.^20,33,34^ In the current study, recurrence was classified into seven categories on the basis of metastatic site: lung, brain, bone, lymph node, liver, pleura, and adrenal gland (Fig. 7a). We first compared the main driver genes in LUAD patients with or without disease recurrence for at least 5 years during follow-up. *TP53* had a significantly higher mutation frequency in LUAD patients who experienced disease recurrence after surgery (49% vs 29%, Fig. 7b). *KMT2C* (6% vs 2%) and *CTNNB1* (5% vs 2%) were also more frequently mutated in patients with disease recurrence. Regarding SCNAs, the frequencies of gains in chromosomes 7p, 5p, and 7q and of loss in chromosomes 21p and 9p were higher in patients with disease recurrence (Supplementary information, Fig. S15a). For specific organs, we found that *TP53* mutations were significantly correlated with brain metastasis, and mutations in genes of the TP53 pathway were significantly correlated with brain and lung recurrences (Fig. 7c). Several SCNAs were also associated with site-specific recurrence: gains of 7p and 8q and loss of 9p with bone recurrences; gains of 7p, 12q, and 14q with brain recurrences; gains of 1q and 7p with lymph node metastasis; and gain of 8p with pleural metastasis (Fig. 7d). Moreover, high APOBEC signature activities were more commonly seen in patients with brain metastasis (Fig. 7e).

To understand the transcriptomic changes in patients with site-specific recurrences, we compared transcriptomic profiles and identified DEGs between patients with and without recurrences during follow-up. Metastatic patients had five significantly upregulated genes, including *SPP1*, and four significantly downregulated genes, including *SFTPC*, compared with non-metastatic patients (Supplementary information, Fig. S15b). In site-specific comparisons, both *SPP1* upregulation and *SFTPC* downregulation were observed in patients with brain metastasis, and *SPP1* upregulation was also noted in patients with bone and lung recurrences (Supplementary information, Fig. S15b). Taken together, these results reveal that patients with site-specific metastasis after surgery exhibited unique genomic and transcriptomic profiles.

We next explored whether tumors harboring different genetic changes influenced the time to metastasis. We found that patients with alterations in the NRF2 pathway had the shortest time to disease relapse (Fig. 7f). For specific metastatic sites, alterations in the NRF2 pathway, along with mutations in *TLR4*, *FAT1*, and *CUL*3, were associated with a shorter time to relapse for brain metastasis (Fig. 7g). In terms of SCNAs, loss of chromosome 13q34 was associated with a shorter time to bone metastasis. Gains in chromosomes 7p (11.2, 14.3, 15.2, 21.1) and 14q (12, 13.1, 13.2, 13.3, 21.1), as well as losses in chromosomes 8p (23.1, 23.2) and 9p (21.3) were associated with a shorter time to brain metastasis, and gains in chromosomes 1q (21.1, 21.3, 23.1, 23.3), 8q (22.1, 22.2, 22.3), and 14q (12, 13.1, 13.2, 13.3) were associated with a shorter time to metastasis in lung recurrence (Fig. 7h).

To further explore the prediction of LUAD relapse, we developed a model integrating relapse-related molecular events and clinical information. Transcriptomic and genomic features associated with LUAD relapse, along with clinical phenotypic variables, were used to train a predictive model using the LightGBMXT method in the AutoGluon platform. The integrative model combining omics and clinical data achieved an area under the curve (AUC) of 0.89 on the testing set, compared with 0.85 for the omics-only model and 0.84 for the clinical-only model (Supplementary information, Fig. S16).

## Discussion

Cancer is a systemic disease whose initiation and progression are a multistep process.^35^ In this study, we analyzed the genomic and transcriptomic profiles of 1008 LUAD samples from 954 patients across different pathological and radiological stages. Our aim was to provide a comprehensive understanding of the genomic and transcriptomic changes that occur as LUAD progresses from pre-invasive stages to late-stage metastatic tumors. Driver mutations in major oncogenes in LUADs (e.g., *EGFR, KRAS, ERBB2*) were identified as early as the pre-invasive stages, namely AAH, AIS, and MIA, indicating that cells had already acquired genetic changes and undergone malignant transformation at these very early stages. From a clinical perspective, these pre-invasive LUADs, along with LUADs that presented as pure GGOs on CT imaging, had nearly 100% 5-year or even 10-year survival rates after complete surgical resection.^6–9^ As the survival rate drops dramatically when tumors progress to invasive stages, this indicates a curative time window for complete surgical resection in patients with early-stage LUAD.^15,36^ Therefore, the identification of key molecular events during LUAD progression will help to define this curative window more precisely, enabling recognition of alarms at an early stage while avoiding overtreatment.

Previous studies have depicted the mutational landscapes and identified the major driver genetic events in LUADs.^11–14^ However, there is a lack of studies focusing on dynamic changes in the genetic background as LUAD progresses. In this study, *EGFR* mutations were detected in 64.5% of sequenced samples, reflecting the East Asian population and the predominance of never-smokers in the study cohort. Compared with invasive LUADs, pre-invasive stages had lower mutation frequencies of genes in the RTK-RAS pathway. Moreover, mutations in tumor suppressor genes were more frequently detected in invasive LUADs, suggesting that loss of function of these genes plays a key role in the progression of LUAD. Specifically, *TP53* was detected in 1% of AAH/AIS/MIA samples but in 32% of LUAD samples, highlighting its critical role in disease progression from precursors to invasive tumors.^37,38^ In line with a previous study that involved 98 pre-invasive and 99 invasive LUADs, we found that TMB, APOBEC signature activity, WGD events, and SCNA burden all increased as tumors progressed from pre-invasive to invasive stages.^22^ Using WGS data, we were able to detect SVs, revealing that SVs were more prevalent at higher disease stages. A sharp increase in SV burden from MIA to stage I LUAD suggested a sudden increase in genomic instability as the disease progressed from pre-invasive to invasive stages.

A similar trend was observed radiologically, offering insights into the genomic changes between pure/mixed GGOs and solid nodules on CT scans.^6,7^ With the widespread use of CT scanning, an increasing number of pure and mixed GGOs have been incidentally identified.^39^ It has been reported that patients with pure GGOs have an excellent survival rate after surgery.^8,9^ As LUADs may develop from pure GGOs to mixed GGOs and ultimately to solid nodules, our study provides insights into the genomic and transcriptomic changes during this process, offering evidence for the curative window of LUAD.

Although oncogenes are usually considered mutually exclusive, their co-occurrence with tumor suppressor genes has been reported to be associated with poor prognosis.^11,30,40^ In this study, we analyzed the prognostic value of co-mutations involving one oncogene and one tumor suppressor gene or two tumor suppressor genes. Using WGS data and follow-up information, we identified several pairs of co-mutations that had prognostic value. Among them, co-mutations involving *TP53* were the most common and had the most significant negative effect on survival, suggesting a pivotal role for *TP53* in LUAD. Interestingly, mutual exclusivity was also observed between oncogenes and tumor suppressor genes; for example, *EGFR* was mutually exclusive with *STK11* and *KEAP1*, and *BRAF* was mutually exclusive with *TP53*. As co-mutations have been reported to affect immunotherapy outcomes in non-small cell lung cancer, these findings could shed light on individualized treatment strategies for LUAD.^41,42^

Driver mutations are those that play critical roles in cancer initiation and progression.^43,44^ The identification of driver mutations is significant for the discovery of novel drug targets, although defining a driver mutation can often be challenging.^45,46^ In this study, we identified a hotspot mutation (p.E102–I103 deletion) in *MAP2K1*, which encodes a protein kinase essential for the MAP kinase signal transduction pathway.^47^ This mutation was previously found in 2 out of 230 (0.9%) patients with LUAD.^11^ Interestingly, in our study cohort, 13 patients harbored this mutation, with 11 (6.9%) of them detected in pre-invasive samples. This mutation hotspot was also detected in our previous study, where it was present in 4 out of 98 (2.0%) pre-invasive adenocarcinoma patients.^22^ To assess the driving potential of this mutation, we performed in vitro and in vivo experiments and found that ATII organoids overexpressing *MAP2K1*^*ΔE102−I103*^ significantly outgrew ATII organoids overexpressing WT *MAP2K1* and those in the control group. Moreover, in vivo experiments suggested that *MAP2K1*^*ΔE102−I103*^ in the absence of *Trp53* could drive LUAD formation in NSG mice. These results suggest that the MAP2K1 p.E102–I103 deletion could be a novel driver mutation of pre-invasive adenocarcinoma. Further experiments indicated that *EGFR* and *KRAS* mutations confer a stronger selective advantage compared with the *MAP2K1* mutation. The potential reasons are as follows: (1) the *MAP2K1* mutation has reduced oncogenic strength compared with *EGFR* or *KRAS* mutations, perhaps because MAP2K1 occupies a downstream position in the signaling cascade, and upstream alterations (such as *EGFR* or *KRAS* mutations) may exert broader oncogenic effects; (2) the mutual exclusivity between *MAP2K1* mutation and *EGFR* or *KRAS* mutations likely reflects functional redundancy within the RAS/RAF/MEK/ERK signaling axis, resulting in selective pressure to retain only one active oncogenic event in this pathway per tumor clone; (3) the absence of *MAP2K1* mutation in invasive adenocarcinoma may suggest an evolutionary bottleneck, whereby only clones with stronger proliferative or survival advantages can successfully expand and progress. In addition, we identified SM1-71 as a potential inhibitor of *MAP2K1*-mutated LUAD. Collectively, these findings suggest that *MAP2K1* mutations might be early clones in LUAD and a potential therapeutic target for pre-invasive adenocarcinoma.

Metastasis is a leading cause of cancer-related death, and LUADs harboring different genomic alterations exhibit organotropism.^20,48^ In this study, we compared the genomic and transcriptomic profiles of patients with site-specific metastasis to those without metastasis after surgery. We found that *TP53* mutations were significantly associated with brain metastasis and that mutations in the TP53 pathway were significantly associated with brain metastasis and lung recurrence. High APOBEC signature activity was observed in patients with brain and lymph node metastasis. Upregulation of *SPP1* and downregulation of *SFTPC* were also found in patients with metastasis, both in general and specifically in the brain, and *SPP1* was also upregulated in patients with lung and bone recurrences. The increased expression of *SPP1* may originate from both tumor cells and tumor-associated macrophages (TAMs). Prior studies have shown that SPP1 promotes invasion and metastasis by targeting COL11A1 in malignant tumor cells.^49,50^ Emerging evidence further suggests that SPP1^+^ TAMs can remodel the tumor microenvironment and facilitate tumor metastasis,^51,52^ consistent with the observed association between *SPP1* expression and Treg infiltration in our study. Our findings indicate that SPP1 could be a potential biomarker for monitoring the progression of LUAD. SFTPC, a marker of ATII cells, has been reported to inhibit epithelial-to-mesenchymal transition by upregulating SOX7 and inactivating the WNT/β-catenin pathway.^53^ Another study demonstrated that *SFTPC* expression is progressively downregulated during lung cancer progression and is completely absent in lung cancer tissues with brain metastases.^54^ Despite these findings, the molecular mechanisms underlying metastasis in LUAD require further investigation.

One limitation of this study is its single-center, retrospective design. In addition, only 10 stage IV LUAD patients were included, as they were accidentally found to have pleural dissemination during surgery. However, with its large sample size, comprehensive clinical, pathological, and follow-up data, and the use of both WGS and RNA-seq techniques, we hope that this study provides valuable insights into the dynamics of genomic and transcriptomic profiles during the progression of LUAD.

**Fig. 8:**
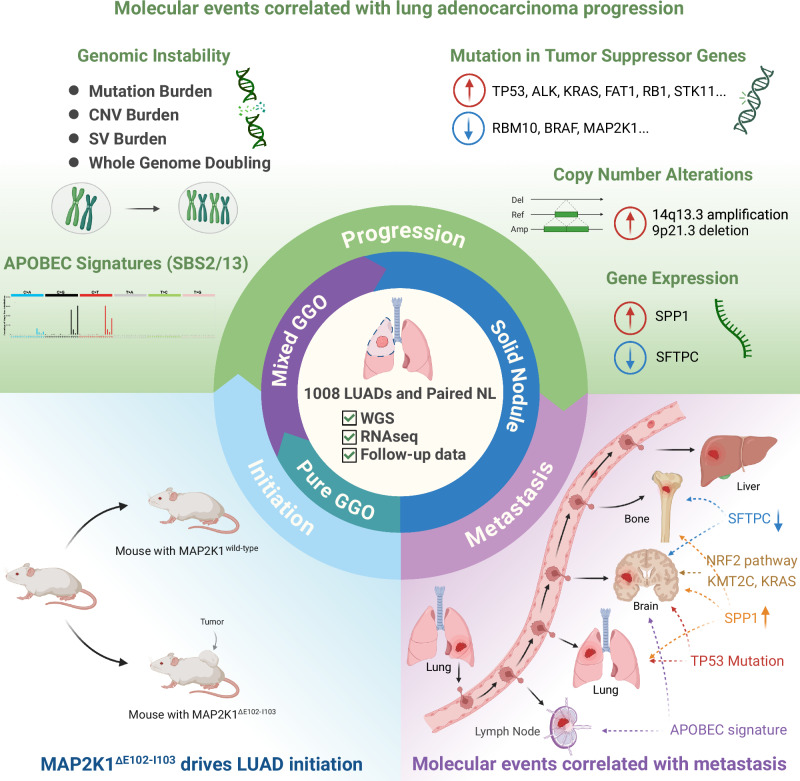
Summary of the study. Analysis of 1008 LUAD samples identified the MAP2K1 E102–I103 *deletion* as a potential driver of LUAD initiation. LUAD progression was associated with increased genomic instability, including a higher tumor mutational burden, increased SCNA burden, and increased structural variations. Furthermore, patients with site-specific recurrences exhibited distinct genomic and transcriptomic profiles.

In summary, this large-cohort, single-institution genomic and transcriptomic study provides a comprehensive understanding of the progression of LUAD. Key molecular events were identified at different evolutionary stages, from tumor initiation to late-stage disease with distant metastasis (Fig. 8). This study can help to define the curative window for surgical resection, guiding individualized treatment strategies and offering evidence for drug discovery in LUAD.

## Materials and methods

### Study cohort

A total of 1008 surgically resected samples from 954 patients with LUAD who underwent surgery between August 2011 and March 2019 at the Department of Thoracic Surgery, Fudan University Shanghai Cancer Center, were retrospectively included in this study in accordance with the Declaration of Helsinki. None of the patients received neoadjuvant therapy. Informed consent from all patients for sample donation to the tissue bank of Fudan University Shanghai Cancer Center was obtained from patients themselves or their relatives. This study was approved by the Committee for Ethical Review of Research (Fudan University Shanghai Cancer Center Institutional Review Board, No. 2301268-3).

### Radiological and histological evaluation

Each patient received whole-lung CT scanning before surgery. Tumor size on CT images was defined as the maximum diameter of the lesion, and the solid component on the single largest axial dimension was recorded on the lung window. Based on the CT images, these pulmonary nodules were further categorized into 3 groups: pure GGOs, in which there was no solid component in one pulmonary nodule; mixed GGOs, in which both solid and GGO components were present in the same pulmonary nodule; and solid nodules, in which the nodule contained only solid components (Fig. 5a). CT images were reviewed by two independent radiologists, with inter-observer and intra-observer agreements measured to quantify their reproducibility and accuracy, as described previously.^6^

For histological diagnosis, intraoperative frozen-section diagnosis was first made after the tumor was resected, and final postoperative pathological diagnosis was made by two independent pathologists after surgery. Following IASLC/ATS/ERS guidelines, tumors were classified as AIS, MIA, or invasive LUAD on the basis of their histological presentations.^55^ Invasive LUADs were further classified into different subtypes, namely lepidic, acinar, papillary, micropapillary, solid, and invasive mucinous subtypes. The subtypes were determined according to cell morphology under the microscope and were recorded in 5% increments. The predominant subtype was defined as that with the largest percentage in one sample.^55^ The pathological stage of the disease was determined according to the eighth edition of the TNM staging system.^10^

### Follow-up protocol

Patients were followed up regularly after surgery as described previously.^6^ In brief, patients were followed up every 3 months for the first 2 years after surgery, during which time physical examinations, chest CT scans, and abdominal ultrasonography were performed every 3–6 months. The follow-up interval was then changed to every 6 months for the third year and once a year from the fourth year onwards. Brain CT or magnetic resonance imaging (MRI) and bone scintigraphy were performed every 6 months for patients with invasive LUADs in the first 3 years. In addition, positron emission tomography (PET)-CT scans were performed if necessary. RFS was defined as the time between surgery and the first recurrence or last follow-up. Patients with no recurrence who died from other causes were censored on that date. OS was defined as the time between surgery and death or the last follow-up date.

### WGS and RNA-seq

Genomic DNA from tumors and paired adjacent normal lung tissues was extracted and prepared using the QIAamp DNA Mini Kit (Qiagen, Germany) following the manufacturer’s instructions. A total of 2 μg genomic DNA for each sample was fragmented into an average size of ~350 bp. Libraries were constructed and sequenced on the Illumina NovaSeq 6000 platform to obtain 150-bp paired-end reads. Total RNA from tumors and paired adjacent normal lung tissues was extracted and prepared using NucleoZOL (Macherey-Nagel, Germany) and NucleoSpin RNA Set for NucleoZOL (Macherey-Nagel, Germany) following the manufacturer’s instructions. The total RNA from each sample was used as the initial material for RNA sample preparation. Ribosomal RNA was removed using the KAPA Stranded RNA-Seq Kit with RiboErase (KK8481, Roche, Switzerland). Libraries were generated and sequenced on the Illumina NovaSeq 6000 platform to obtain 150-bp paired-end reads.

### Quality control

To ensure quality control throughout the entire process of generating sequencing data, we relied on the Quartet reference materials.^56,57^ We performed DNA and RNA library construction with a maximum of 96 samples: 94 lung cancer samples and 2 Quartet reference samples. This design guaranteed that each lung cancer sample was accompanied by Quartet reference samples throughout the processes of library construction, sequencing, and data analysis.

Our prior studies established quality-control metrics based on Quartet reference materials, including the Mendelian concordance rate (MCR) and F1 score at the DNA level^58^ and the signal-to-noise ratio (SNR) at the RNA-seq level.^59^ Once all batches of lung cancer samples and their corresponding Quartet samples were generated, we analyzed the Quartet samples and calculated the MCR, F-score, and SNR. These metrics were then used to assess the quality of each batch of data. Simultaneously, we monitored the consistency of experimental conditions across batches, thereby guaranteeing the generation of high-quality sequencing data.

The quality and amount of genomic and transcriptomic sequencing data were assessed using FastQC (v0.11.9). Fastq Screen (v0.15.1) software was used to analyze the ratio of sequenced reads across species to determine whether the target DNA and RNA were contaminated with genetic material from other species. NGSCheckMate (v1.0.0)^60^ was used to calculate the allele fractions of known single-nucleotide polymorphisms (SNPs) in each sample and to assess whether the samples originated from the same individual. This was done by comparing the correlation of allele fractions of these SNPs between samples. In cases where tumor and normal samples were unpaired, they were excluded before somatic variant calling.

The amount of WGS data for this cohort exceeded 150 GB, and the amount of RNA-seq data exceeded 15 GB (Supplementary information, Fig. S17a, b). NGScheckmate confirmed that both the tumor and paired normal samples originated from the same individual (Supplementary information, Fig. S17c). All samples underwent base-quality assessment, including analysis of Q30 scores (Supplementary information, Fig. S17a, b), with Quartet DNA and RNA standards incorporated into each batch for quality control (Supplementary information, Fig. S17d). Each batch included 94 LUAD samples and 2 Quartet standard material samples. The quality of Quartet DNA and RNA data was evaluated using specific metrics: F-score and MCR for DNA sequencing and SNR for RNA sequencing. The results of these evaluations — F-score, MCR, and SNR — demonstrated that the WGS and RNA-seq data generation for all tumor samples in this cohort were consistently reliable throughout the entire process (Supplementary information, Fig. S17e, f).

### Somatic variant analysis

We harnessed the capabilities of the BWA algorithm, integrated into Sentieon (version sentieon-genomics-202112.04),^61,62^ to align reads originating from tumor and normal samples to the human reference genome (GRCh38.d1.vd1.fa). Following this alignment, Sentieon’s Dedup function was used to tag and eliminate duplicate reads, ensuring the integrity of downstream analyses. For subsequent analyses, including somatic variant and indel assessments, we used the aligned data from both tumor and normal samples (Dedup BAMs). The comprehensive Mutation Variant Calling process involved the use of TNseq,^61^ TNScope^62^ (sentieon-genomics-202112.04), and strelka2 (v2.9.10).^63^ Variants that received concurrent calls from at least two software tools were retained. In addition, we performed manual verification using the Integrative Genomics Viewer (IGV) for common mutation sites in LUAD. Somatic variants were annotated using ANNOVAR (v2019-10-24)^64^ and Variant Effect Predictor (VEP, v104.0).^65^ The ClinVar pathogenicity information, SIFT, and PolyPhen-2 results were derived from the annotation section of VEP. TMB was characterized as the total number of non-synonymous single-nucleotide variants per megabase (mut/Mb), with the capture size aligning with the TCGA cohort.

### Somatic variant signatures

Mutational signatures were analyzed using the SigProfiler computational framework,^66^ with SigProfilerExtractor (v1.1.21) used to profile SBS. SBS, also known as single-nucleotide variants, involve the replacement of specific nucleotide bases. In the context of pyrimidines within the Watson–Crick base pair, six possible substitutions exist: C>A, C>G, C>T, T>A, T>C, and T>G. A total of 96 COSMIC SBS mutations have been identified. The mutations in each sample were decomposed based on the COSMIC SBS mutation signature (v3.3) using the Analyzer function in SigProfilerExtractor. Subsequently, the mutation probability or number of mutations for each SBS signature in each sample was generated, enabling diverse genetic characterizations based on clinical phenotype. APOBEC-induced mutations are primarily linked to C>T transition events. In addition to SBS2 and SBS13, we also used the trinucleotideMatrix function in maftools to evaluate the APOBEC Enrichment Score.

### Copy number variant analysis

The parsing of copy number variants was executed using ascatNgs (v4.3.3)^67^ with the ASCAT (v2.5.1)^68^ wrapper. Subsequent copy number variant analyses were performed on tumor and normal BAM files derived from Sentieon-BWA alignment. The reference genome used for these analyses corresponded to the human reference genome used in the Sentieon-BWA alignment (GRCh38.d1.vd1.fa). In the clinical phenotype linkage analysis, samples that lacked ASCAT solutions or demonstrated 100% purity were excluded from both the copy number and clinical phenotype linkage analyses.

GISTIC2 (v2.0.23)^69^ was used to analyze copy number variation (CNV) amplification or deletion changes at the arm, focal, and gene levels. The CNV segment file for each sample was obtained using ascatNGS, and these segment results were used as input for GISTIC with the following parameters: ta 0.25, td 0.25, qvt 0.25, cap 1.5, brlen 0.5, conf 0.95, armpeel 1, broad 1, and savegene1. In the cohort, significant changes in arm events were identified using a threshold of *q* < 0.01, and deletions less than −1 and amplifications greater than or equal to 2 were displayed in the oncoplot. At the gene level, copy number alteration (CNA) amplification and deletion were identified using thresholds of −2 and +2. The TCGA CNV ASCAT2 data were analyzed using the same methods and parameters.

GISTIC amplification and deletion profiles were independently analyzed using the R package cngpld (v0.1), which uses a Gaussian process latent difference model. This approach enabled the identification of specific genomic regions within the invasive LUAD cohort that exhibited significantly higher G scores compared with those in the pre-invasive LUAD cohort.

### HLA LOH

HLA LOH identification was performed using the LOHHLA algorithm.^24^ First, the HLA typing for each patient was determined using POLYSOLVER (v1.0.0)^70^ based on WGS sequencing data from paired normal samples. Subsequently, the HLA fasta file was compared with the WGS data from tumors and paired normal tissues for each patient to evaluate the LOH status of their HLA typing.

### SV analysis

SV analysis was performed on aligned tumor and normal BAM files using GRIDSS2 (v2.13.2).^71^ Following the retrieval of SV data from all tumor samples, the events were filtered using the panel-of-normal file provided by GRIDSS2. The SV events from each sample were then categorized into five types based on variant characteristics: CTX, Inversion, Insertion, Deletion, and Duplication. SV signatures were constructed using SigProfilerExtractor (v1.1.21)^66^ with default parameters. Chromothripsis was identified using ShatterSeek.^72^

### RNA-seq data analysis

Raw FASTQ data were used for expression profiling with the HISAT2–StringTie pipeline.^73^ Initial preprocessing steps involved the use of fastp (v0.36)^74^ to remove adapters from the raw RNA-seq reads. Reads were aligned to the human reference genome (GRCh38, release-84) using HISAT2 (v2.2.1), with data sourced from the Genomic Data Commons (GDC). Following alignment, the reads were assembled into transcripts or genes with StringTie (v2.2.1) using the genome annotation file (gencode.v36.annotation.gtf). Gene expression was quantified as fragments per kilobase of exon model per million mapped fragments (FPKM). To address potential confounders in the RNA-seq analysis, propensity score matching was performed for RNA integrity number and tumor purity, and key transcriptomic changes associated with tumor progression remained significant (Supplementary information, Fig. S18). Sequencing of the Quartet reference samples also indicated no apparent confounding effects in the RNA-seq data.

### Gene fusion detection

For analysis of gene fusions, STAR-Fusion^75^ was used to identify and profile potential fusion events in the RNA-seq data. The workflow involved aligning RNA-seq reads to a reference genome, systematically examining the aligned reads for abnormal gene connections indicative of fusion events, and implementing rigorous filtering criteria to preserve only validated fusion events for subsequent analysis. The confirmed fusion events then underwent detailed annotation, furnishing comprehensive insights into the implicated genes, their genomic coordinates, and potential functional implications.

### Differential gene expression

The limma^76^ R package (v3.50.0) was used for differential expression analysis comparing clinical phenotypes. DEGs were identified using standard cutoffs (*p* < 0.05, |log_2_(fold change)| ≥ 1).^77^ Fisher’s exact test (*p* < 0.05) was used to identify differentially mutated genes between GGOs and solid cohorts, as well as between the no-relapse and relapse cohorts.

### Immune cell deconvolution

To estimate the relative infiltration of immune cell subsets, we used CIBERSORTx^32^ with bulk RNA-seq expression data as input. Gene expression matrices were normalized to FPKM prior to analysis. The LM22 matrix was used as the reference, and the algorithm was run with 1000 permutations to determine statistical significance of the deconvolution.

### ssGSEA analysis

To estimate gene-set enrichment scores, the R package GSVA (v1.42.0)^78^ was used with the default ssGSVA method. Following the analytical approach of a previous study,^79^ the hallmark gene set (h.all.v7.5.symbols.gmt) was downloaded from the GSEA website.^80^ Enrichment scoring for each sample per hallmark gene set was performed using GSVA, with the method parameters set to ssgsea. Identification of hallmarks that were significantly enriched with genes from the input set was based on an adjusted *p*-value (*p* < 0.05). Differences among the pure GGO, mixed GGO, and solid groups were assessed by ANOVA. The *p*-values were corrected for FDR, and hallmarks with an FDR-adjusted *p*-value of less than 0.05 were selected for further analysis.

### Analysis of molecular events related to recurrence and site of recurrence

Patients who did not experience recurrence for over 5 years were defined as non-recurrent, whereas those who experienced recurrence during follow-up were classified as recurrent. For patients with recurrence, the site of recurrence was documented in as much detail as possible on the basis of CT. We performed a comprehensive analysis of recurrence, along with gene mutations and copy number variants associated with the site of recurrence, using Fisher’s exact test (*p* < 0.05). Gene mutations and copy number variants were analyzed for their association with time to relapse or site-specific relapse using Cox regression (*p* < 0.05). The *q*-value represents the *p*-value after FDR correction. Since multiple genes with CNVs on a chromosome cytoband may be relevant to relapse, relapse site, or relapse timing, we present only the chromosome cytoband where these genes are located in the final presentation.

### Development of a predictive model for tumor relapse

At the gene expression level, DEGs associated with pathological stage, radiological stage, and metastasis were identified through differential expression analysis, whereas relapse-related mutation events were obtained at the mutation level. Clinical information primarily included pathological stage, radiological stage, age, gender, and smoking history. The entire cohort was divided into training and testing sets in a 7:3 ratio. The model was designed to predict tumor relapse. Three predictive models were constructed for the training set using the TabularPredictor module of AutoGluon^81^: combined omics and clinical information, omics alone, and clinical information alone. After model training, the best-performing model was selected for validation with the testing set. The AUC was calculated for each model.

### Statistical analysis

All statistical analyses were performed using R (v4.1.2). Various statistical tests, including *t*-tests, Fisher’s exact tests, and Pearson correlations, were applied. Kaplan–Meier survival analysis and Cox regression hazard modeling were performed using the R packages survival (v3.2-13) and survminer (v0.4.9). Kaplan–Meier survival analysis, coupled with the log-rank test, was used for both OS and RFS assessments. Heatmaps were generated using the R package ComplexHeatmap (v2.15.4).^82^ Principal component analysis was performed with the R package stats (v4.1.2). Oncoplots and lollipop plots were created using maftools (v2.10.05).^83^ Boxplots and scatter plots were created using the R packages ggpubr (v0.4.0) and ggplot2 (v3.4.0). This comprehensive suite of analyses and visualizations ensures a thorough exploration and presentation of the dataset.

### Mouse studies

All mouse work was approved by the Animal Ethics Committee of the School of Basic Medical Sciences at Fudan University and performed in compliance with the NIH Guide for the Care and Use of Laboratory Animals. *Trp53*^*L/L*^;*Cas9*^*tdTomato*^ mice (*Trp53* homozygous and LSL-Cas9^tdTomato^ heterozygous) were obtained to generate *Trp53*^*L/L*^;*Cas9*^*tdTomato*^ ATII lung organoids by crossing *Trp53* conditional knockout mice with Cas9^tdTomato^ conditional knock-in mice. In brief, LoxP sites were inserted flanking the exon 5–7 region of *Trp53* via homologous recombination. *Trp53* was knocked out by Cre recombinase-mediated removal of *Trp53* exons 5–7 using the Ad-Cre system. NSG (procured from Gem Pharmatech Co., Ltd., Nanjing, China) were used for the human ATII organoid allografts.

### Organoid culture and manipulation

*Trp53*^*L/L*^;*Cas9*^*tdTomato*^ ATII lung organoids were generated from 6–8-week-old *Trp53*^*L/L*^;*Cas9*^*tdTomato*^ mice of the C57BL/6J background. In brief, the lungs were dissected and rinsed twice with phosphate-buffered saline (PBS). The tissues were finely chopped using scissors and then digested in a solution of collagenase D and DNase I in Hank’s Balanced Salt Solution at 37 °C for 30 min. Following incubation, the digested tissue was filtered through a 70-μm cell strainer to obtain single-cell suspensions. The cells were pelleted by centrifugation at 200× *g* for 5 min, resuspended in Advanced DMEM medium, and plated in a 6-cm dish. After 36 h of culture, the cells were washed 5–6 times with PBS and treated with 1 mL of trypsin-EDTA (Meilunbio, Cat# MA0233) to digest epithelial cells. The ATII lung organoids were maintained for successive passages using a 1:2 mixture of PneumaCult Alveolar Organoid Expansion Medium (Stemcell, Cat# 100-0847) and growth factor-reduced Matrigel (Corning, Cat# 354230). *Trp53*^−/−^;*Cas9*^*tdTomato*^ organoids were generated from *Trp53*^*L/L*^;*Cas9*^*tdTomato*^ ATII lung organoids by Ad-Cre virus infection, followed by flow cytometry sorting of tdTomato^+^ cells.

Lentiviral plasmids expressing *MAP2K1* (NM_002755) WT and *MAP2K1* (NM_002755) MT (p.E102–I103del) were constructed by ligating the corresponding PCR products into the MCS-3FLAG-SV40-Cherry-IRES-Blasticidin vector. Lentiviral particles were packaged using the ViraPower Lentiviral Expression System (Thermo Fisher Scientific) following the manufacturer’s manual.

To generate *Trp53*^−/−^;*MAP2K1* WT or *Trp53*^−/−^;*MAP2K1* MT AT organoids, cells were isolated by digesting the Matrigel with 0.25% trypsin-EDTA (Meilunbio, Cat# MA0233) in culture plates for 10 min at 37 °C and washing with PBS. Once the organoids were dissociated, cells were pelleted and resuspended in 50 μL lentiviral solution. Spinoculation was performed by transferring the suspension into a 24-well plate and centrifuging the plate at 600× *g* for 1 h at 32 °C. Plates were then incubated at 37 °C for 6 h before washing the suspension with fresh medium and pelleting the cells to be embedded in fresh Matrigel medium mixture. Antibiotics (blasticidin, 5 μg/mL) were added to the medium to select the infected organoids. Organoid viability was assessed using the bioGenous LivingCell-Fluo Organoid Vitality Assay Kit (bioGenous, Cat# E238004). Morphological images were captured 5 days after passaging under various experimental conditions and analyzed with ImageJ to evaluate the organoid area.

To investigate whether *Trp53*^−/−^;*MAP2K1* MT organoids could form tumors in vivo, 1 × 10^6^ *Trp53*^−/−^;NC or *MAP2K1* WT/MT-overexpressing ATII cells were resuspended in ice-cold 100 μL PBS buffer and implanted subcutaneously into the lower flanks of NSG mice. After tumor formation, tumor volume was estimated every 2–3 days using the following formula: (L × W^2^)/2.

### Ba/F3 co-culture assay

Ba/F3 cells were genetically engineered to express either *MAP2K1*^*ΔE102−I103*^ (mCherry-labeled) or one of two LUAD-associated mutations: *EGFR*^*ΔE746−A750*^ or *KRAS*^*G12D*^ (GFP-labeled). All constructs were verified by Sanger sequencing prior to experimentation. mCherry^+^ *MAP2K1*^*ΔE102−I103*^ cells and GFP^+^ comparator cells (*EGFR*^*ΔE746−A750*^ or *KRAS*^*G12D*^) were co-cultured at a 1:1 ratio in RPMI 1640 medium supplemented with 10% fetal bovine serum and 2 ng/mL IL-3. Three independent replicate experiments were performed for each mutation pair. Cell populations were quantified at day 0, day 3, and day 7 using a CytoFLEX S flow cytometer (Beckman Coulter, FL, USA). Fluorescence signals were detected using 530/30-nm (GFP) and 610/20-nm (mCherry) filters. SM1-71 (MCE, Cat# HY-136848) was used as a MAP2K1 inhibitor.

### IHC staining and analysis

For IHC analysis, paraffin-embedded tissue or organoid sections were first deparaffinized using xylene and then rehydrated through a graded alcohol series into water. Antigen retrieval was performed by heating the sections in citrate buffer (10 mM sodium citrate buffer, pH 6.0) at sub-boiling temperature for 15 min. The sections were then permeabilized with 0.5% Triton-100 in PBS for 20 min. To block endogenous peroxidase activity, a 3% H_2_O_2_ solution was applied for 10 min, followed by three PBS washes. The sections were then incubated with blocking buffer (3% BSA in PBS) for 30 min. Primary antibodies were applied, and the sections were incubated overnight at 4 °C. After three washes, the sections were incubated with the appropriate secondary antibodies for 30 min at room temperature. Signals were visualized using a freshly prepared DAB substrate solution (ZSGB-BIO Company, Beijing, China). Finally, the sections were counterstained with hematoxylin, dehydrated, and mounted with coverslips. The following primary antibodies were used: EpCam (Abcam, Cat# ab71916), TTF-1 (Abcam, Cat# ab76013), and KRT7 (Proteintech, Cat# 17513-1-AP).

### Western blotting and analysis

Western blotting was performed following previously established protocols.^84^ In brief, cells were lysed in RIPA buffer (Meilunbio) containing 50 mM Tris (pH 7.4), 150 mM NaCl, 1% Triton X-100, 1% sodium deoxycholate, 0.1% SDS, and EDTA, supplemented with protease and phosphatase inhibitors (MCE Chemicals, Cat# HY-K0010). A total of 30 μg of protein was loaded onto SDS-polyacrylamide gels, transferred to polyvinylidene fluoride membranes (Millipore), probed with specific antibodies, and visualized.

The following primary antibodies were used: ERK1/2 (1:2000 dilution; Cell Signaling Technology, Cat# 4695), pERK1/2 (1:2000 dilution; Cell Signaling Technology, Cat# 9101), MEK1 (1:2000 dilution; Abcam, Cat# ab32091), pMEK1 (1:2000 dilution; Abcam, Cat# ab96379), and GAPDH (1:5000 dilution; Proteintech, Cat# CL594-60004).

## Supplementary information

Supplementary information, Fig. S1

Supplementary information, Fig. S2

Supplementary information, Fig. S3

Supplementary information, Fig. S4

Supplementary information, Fig. S5

Supplementary information, Fig. S6

Supplementary information, Fig. S7

Supplementary information, Fig. S8

Supplementary information, Fig. S9

Supplementary information, Fig. S10

Supplementary information, Fig. S11

Supplementary information, Fig. S12

Supplementary information, Fig. S13

Supplementary information, Fig. S14

Supplementary information, Fig. S15

Supplementary information, Fig. S16

Supplementary information, Fig. S17

Supplementary information, Fig. S18

## Data Availability

The raw WGS and RNA-seq data have been deposited in the National Omics Data Encyclopedia (NODE) (Accession number: OEP002580) and the Genome Sequence Archive (GSA) (DNA accession number: HRA002624, RNA accession number: HRA002983). Data on CNV, gene expression, and survival prognosis for the TCGA-LUAD cohort were retrieved from the GDC database (https://portal.gdc.cancer.gov/, 2021.02.15).

## Code availability

Code used for the analyses and figures in this study is publicly available (https://github.com/stead99/GTLAD).
