# Supplementary material for: Genomic and transcriptomic dynamics in the stepwise progression of lung adenocarcinoma
Source: Cell Res. 2025 Dec 4;35(12):1037–55. doi: 10.1038/s41422-025-01200-w (PMC12689645; doi:10.1038/s41422-025-01200-w)
Supplement: Supplementary file 2 — Supplementary information, Fig. S2 [file 41422_2025_1200_MOESM2_ESM.pdf]

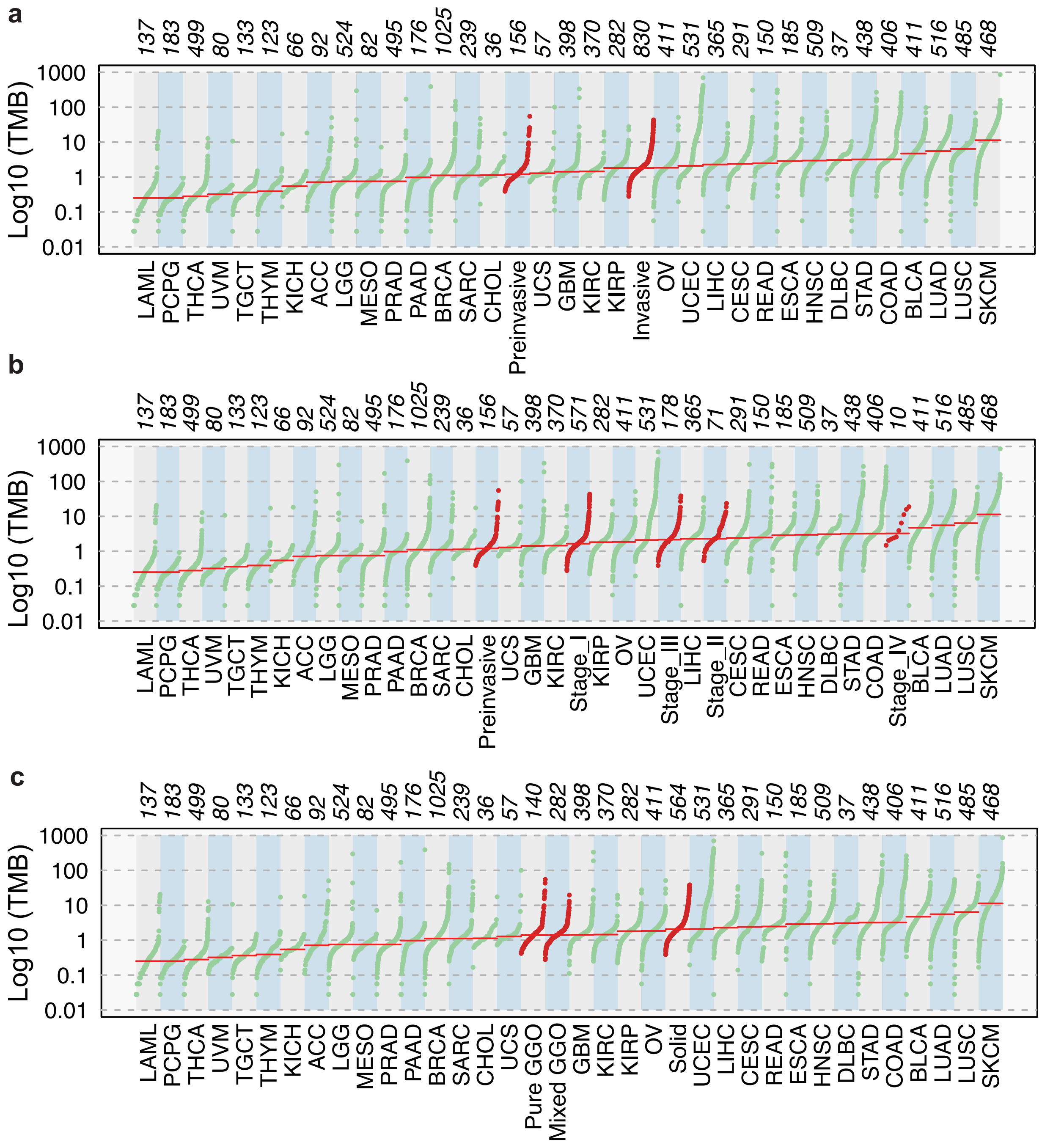

Fig. S2 Comparison of tumor mutation burden among TCGA cohorts and samples in our study cohort divided as preinvasive and invasive ones (a), preinvasive and stage I-IV invasive ones (b), those with different radiological appearances (c).
