# Supplementary material for: Genomic and transcriptomic dynamics in the stepwise progression of lung adenocarcinoma
Source: Cell Res. 2025 Dec 4;35(12):1037–55. doi: 10.1038/s41422-025-01200-w (PMC12689645; doi:10.1038/s41422-025-01200-w)
Supplement: Supplementary file 3 — Supplementary information, Fig. S3 [file 41422_2025_1200_MOESM3_ESM.pdf]

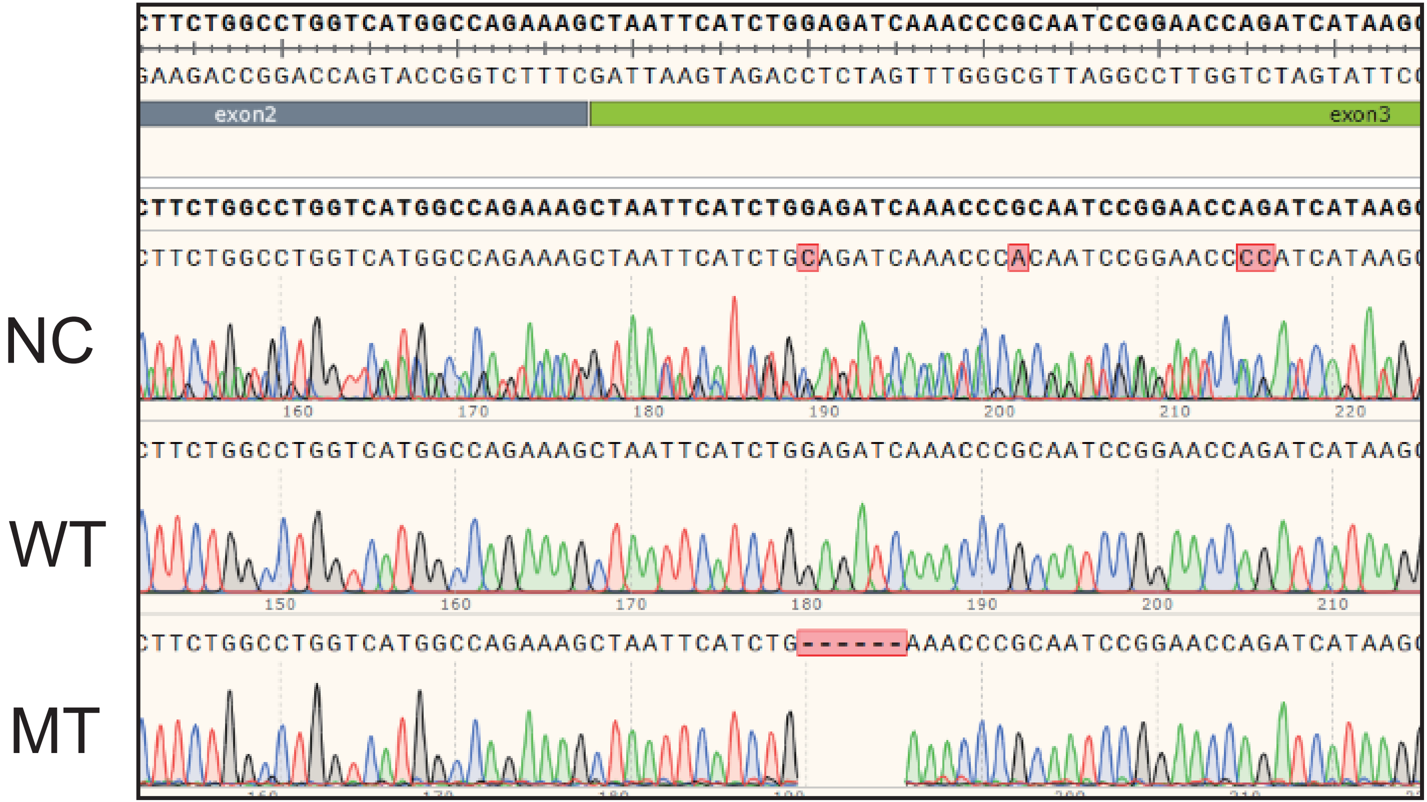

Fig. S3 Sanger sequencing plots showing the mutational status in *MAP2K1* of the 3 groups. NC, the control group; WT, organoids with overexpressed wild-type *MAP2K1*; MT, organoids with overexpressed *MAP2K1*$^{\Delta E102\text{-}I103}$.
