# Supplementary material for: Genomic and transcriptomic dynamics in the stepwise progression of lung adenocarcinoma
Source: Cell Res. 2025 Dec 4;35(12):1037–55. doi: 10.1038/s41422-025-01200-w (PMC12689645; doi:10.1038/s41422-025-01200-w)
Supplement: Supplementary file 4 — Supplementary information, Fig. S4 [file 41422_2025_1200_MOESM4_ESM.pdf]

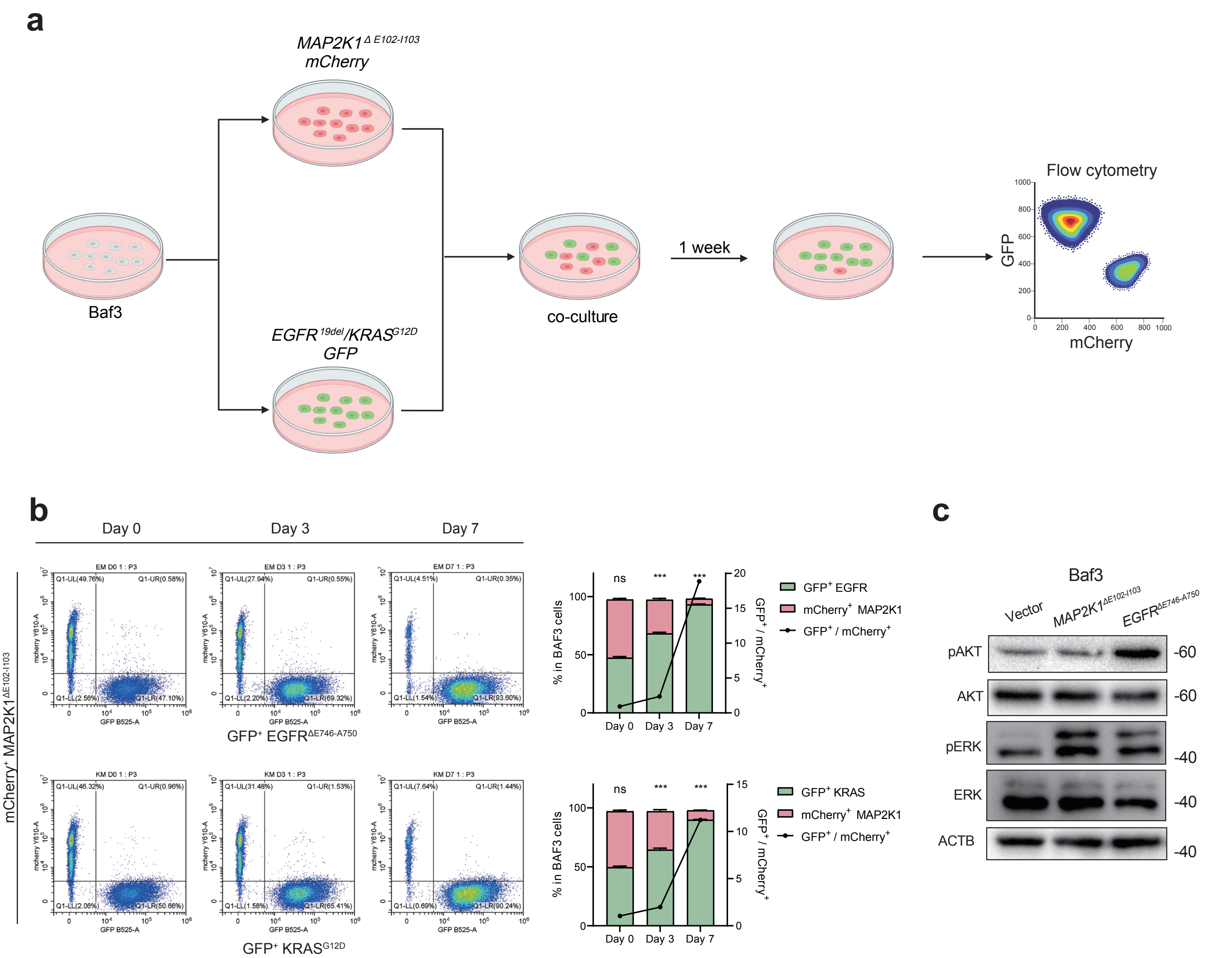

Fig. S4 Comparison of selective advantage conferred by *MAP2K1*$^{ΔE102\text{-}I103}$ versus two major driver mutations in lung adenocarcinoma, *EGFR*$^{ΔE746\text{-}A750}$ and *KRASG12D*. a Study design. MCherry-labeled Ba/F3 cells harboring *MAP2K1*$^{ΔE102\text{-}I103}$ were co-cultured at a 1:1 ratio with GFP-labeled Ba/F3 cells carrying either *EGFR*$^{ΔE746\text{-}A750}$ or *KRASG12D* for 1 week. Flow cytometry was conducted to calculate the proportion of cells carrying different mutations. **b** Left, flow-cytometry of cells harboring different drivers on Day 0, 3 and 7. Right, bar plots demonstrating the proportion of cells harboring *MAP2K1*$^{ΔE102\text{-}I103}$ and *EGFR*$^{ΔE746\text{-}A750}$ or *KRASG12D* on Day 0, 3 and 7. **c** Western blot analysis of phosphorylated and total AKT and ERK in the *MAP2K1*$^{ΔE102\text{-}I103}$, *EGFR*$^{ΔE746\text{-}A750}$, and vector control groups.
