# Supplementary material for: Genomic and transcriptomic dynamics in the stepwise progression of lung adenocarcinoma
Source: Cell Res. 2025 Dec 4;35(12):1037–55. doi: 10.1038/s41422-025-01200-w (PMC12689645; doi:10.1038/s41422-025-01200-w)
Supplement: Supplementary file 5 — Supplementary information, Fig. S5 [file 41422_2025_1200_MOESM5_ESM.pdf]

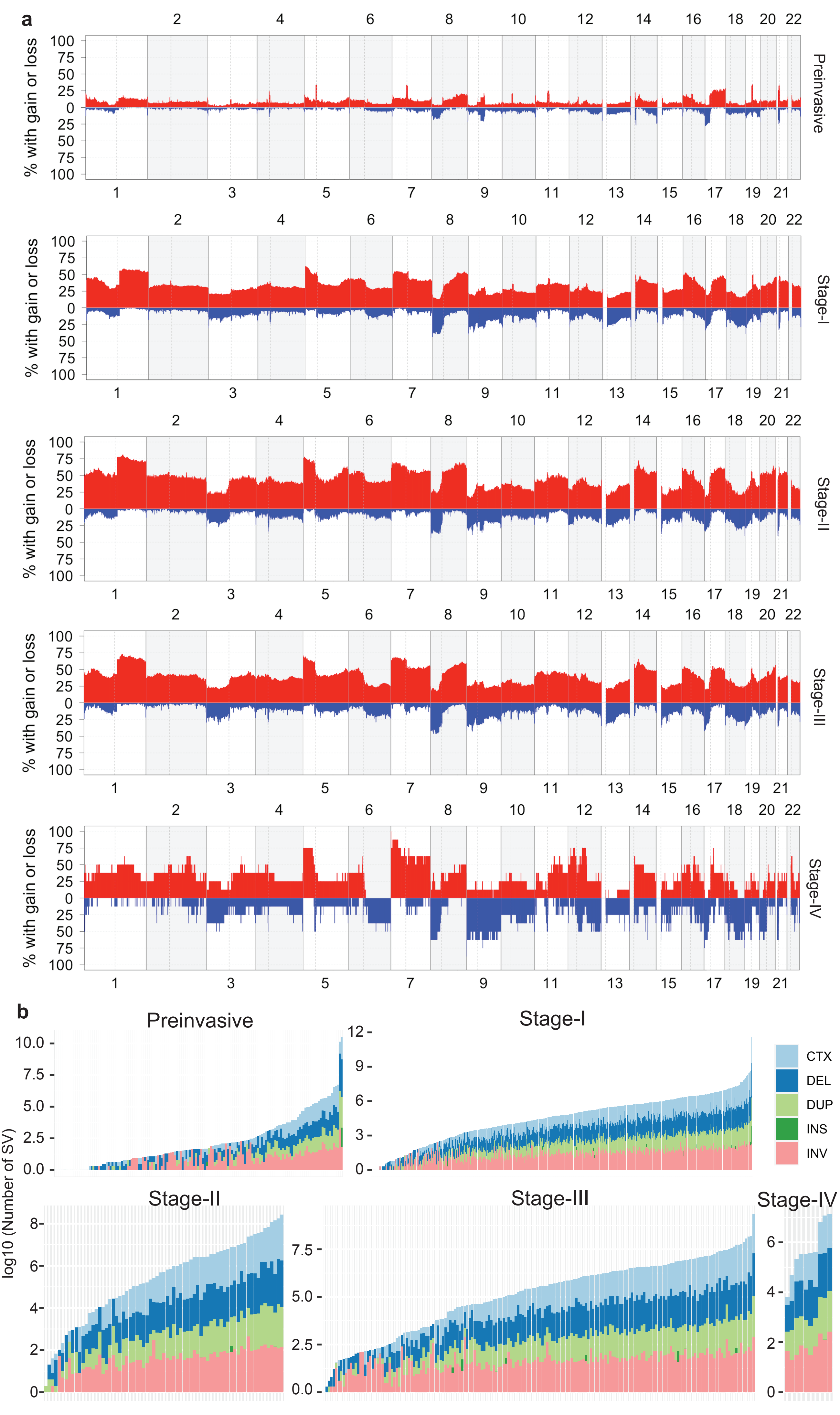

Fig. S5 Overview of somatic copy number alterations (SCNAs) and structural variations (SVs) across different pathological stages of lung adenocarcinoma. a Overview of SCNAs across different pathological stages of lung adenocarcinoma. **b** Overview of SVs across different pathological stages of lung adenocarcinoma.
