# Supplementary material for: Genomic and transcriptomic dynamics in the stepwise progression of lung adenocarcinoma
Source: Cell Res. 2025 Dec 4;35(12):1037–55. doi: 10.1038/s41422-025-01200-w (PMC12689645; doi:10.1038/s41422-025-01200-w)
Supplement: Supplementary file 8 — Supplementary information, Fig. S8 [file 41422_2025_1200_MOESM8_ESM.pdf]

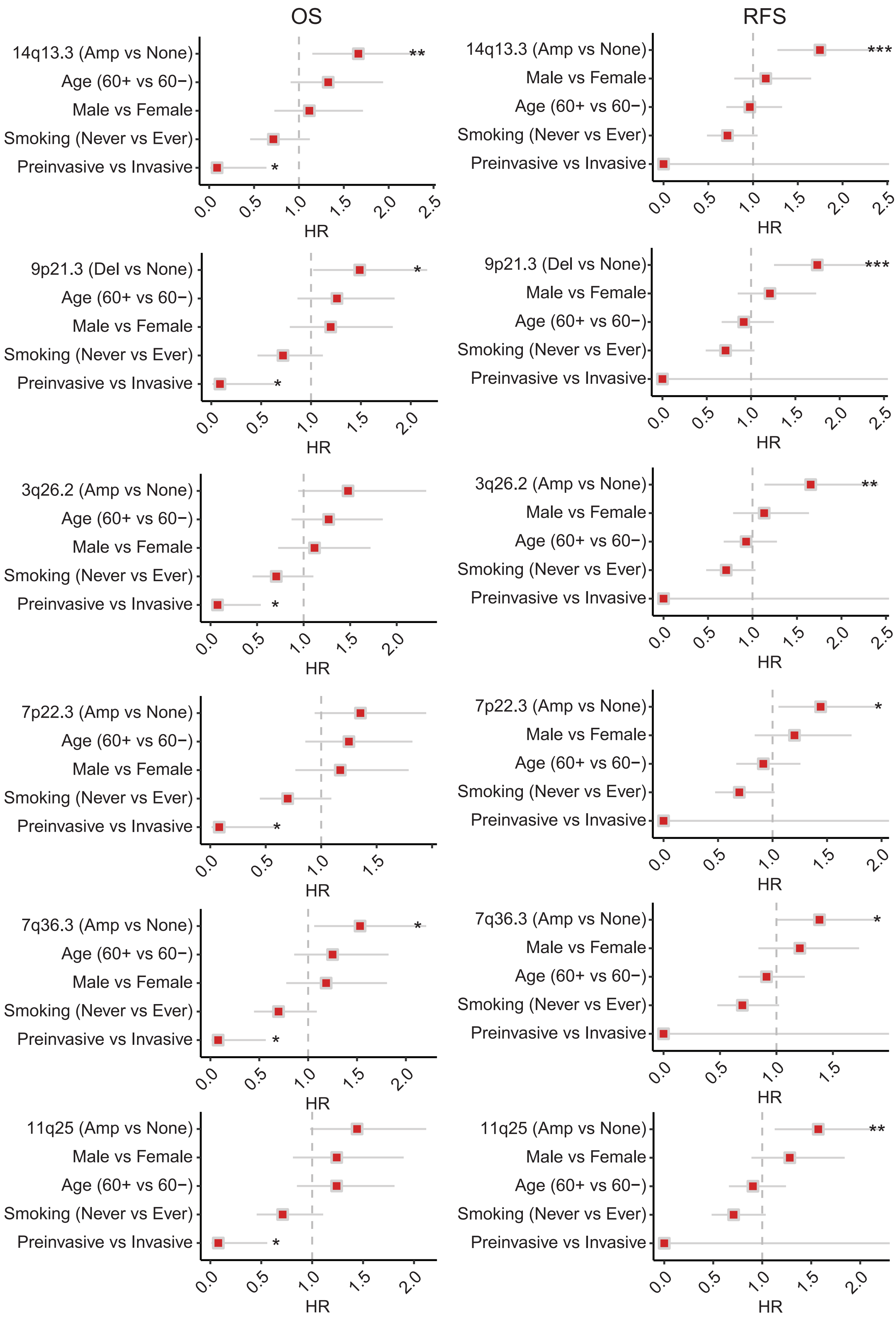

Fig. S8 Survival impacts of focal somatic copy number alterations (SCNAs) using multivariable Cox regression model, excluding the effects of sex, age and smoking status. OS, overall survival; RFS, recurrence-free survival; HR, hazard ratio. Statistical significance was assessed using Cox regression model, * $P < 0.05$, ** $P < 0.01$, *** $P < 0.001$.
