# Supplementary material for: Genomic and transcriptomic dynamics in the stepwise progression of lung adenocarcinoma
Source: Cell Res. 2025 Dec 4;35(12):1037–55. doi: 10.1038/s41422-025-01200-w (PMC12689645; doi:10.1038/s41422-025-01200-w)
Supplement: Supplementary file 9 — Supplementary information, Fig. S9 [file 41422_2025_1200_MOESM9_ESM.pdf]

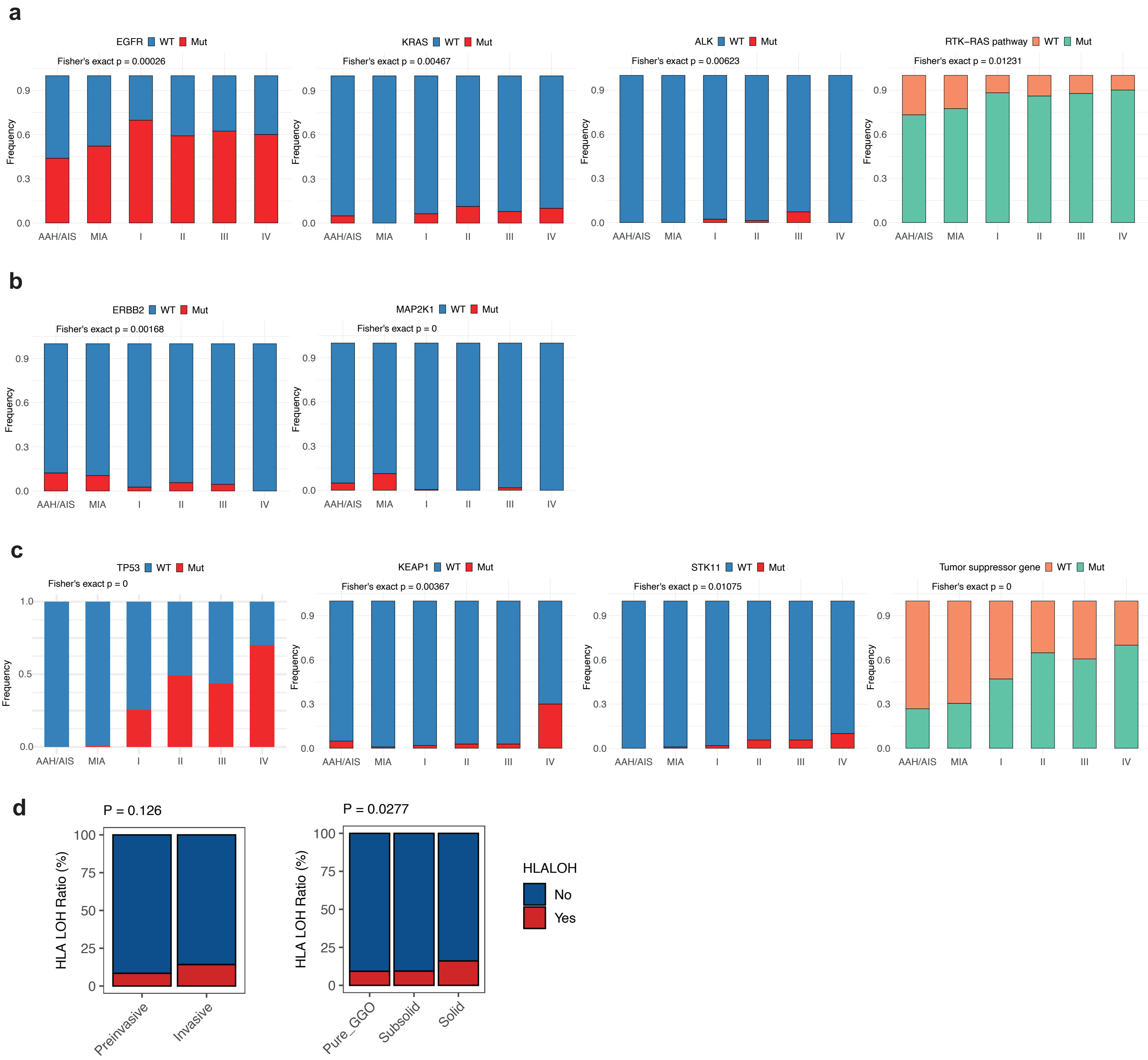

Fig. S9 Comparison of mutation frequencies and HLA Loss of Heterozygosity (LOH) in samples at different pathological stages. a Comparison of *EGFR* and *KRAS* mutations, *ALK* fusions and mutations in the RTK-RAS pathway in samples at different pathological stages. **b** Comparison of *ERBB2* and *MAP2K1* mutations in samples at different pathological stages. **c** Comparison of *TP53, KEAP1, STK11* and tumor suppressor gene mutations in samples at different pathological stages. **d** Comparison of HLA LOH in samples at different pathological and radiological stages. Statistical significance was assessed using *Fisher's exact test*.
