# Supplementary material for: Genomic and transcriptomic dynamics in the stepwise progression of lung adenocarcinoma
Source: Cell Res. 2025 Dec 4;35(12):1037–55. doi: 10.1038/s41422-025-01200-w (PMC12689645; doi:10.1038/s41422-025-01200-w)
Supplement: Supplementary file 10 — Supplementary information, Fig. S10 [file 41422_2025_1200_MOESM10_ESM.pdf]

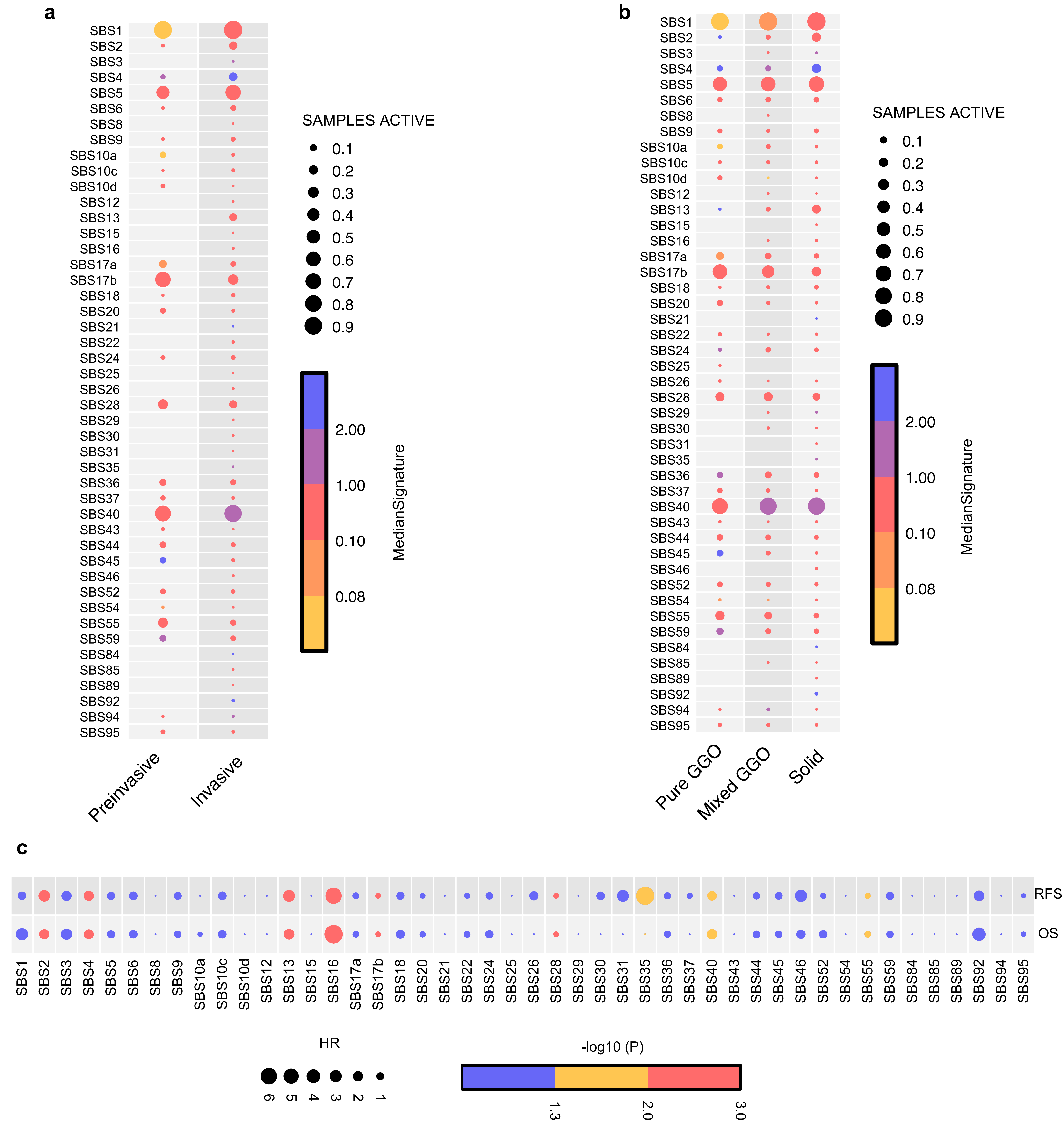

Fig. S10 Analysis of single base substitution (SBS) mutational signatures in lung adenocarcinoma. a Dot plot showing the activity of SBS signatures in preinvasive and invasive samples. **b** Dot plot of SBS signature activity stratified by radiological subtypes. **c** Survival impacts of mutation signatures using multivariable Cox regression model, excluding the effects of sex, age and smoking status. OS, overall survival; RFS, recurrence-free survival; HR, hazard ratio. Statistical significance was assessed using *Cox regression model*.
