# Supplementary material for: Genomic and transcriptomic dynamics in the stepwise progression of lung adenocarcinoma
Source: Cell Res. 2025 Dec 4;35(12):1037–55. doi: 10.1038/s41422-025-01200-w (PMC12689645; doi:10.1038/s41422-025-01200-w)
Supplement: Supplementary file 11 — Supplementary information, Fig. S11 [file 41422_2025_1200_MOESM11_ESM.pdf]

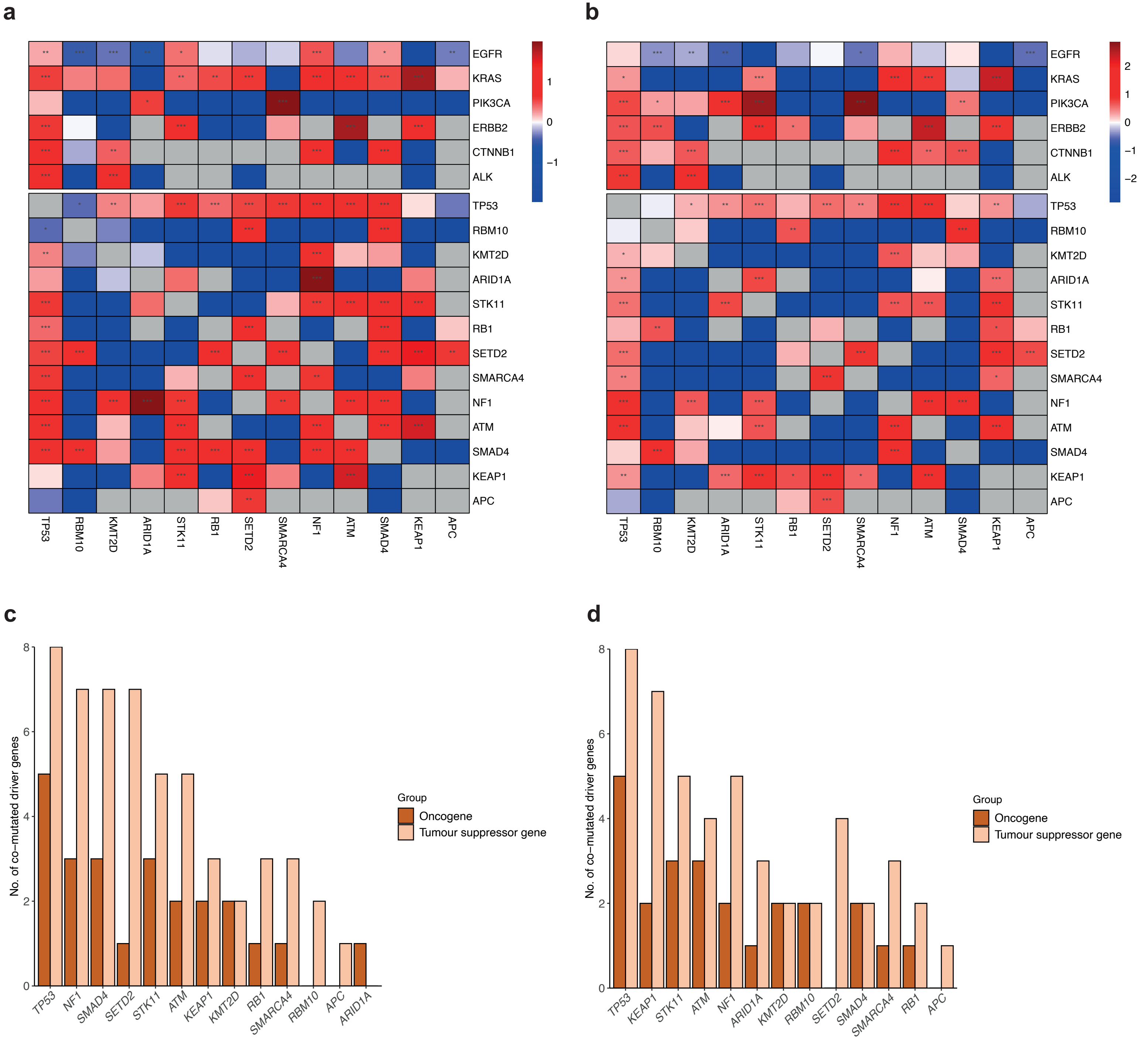

Fig. S11. Survival impacts of co-mutation pairs between oncogenes and tumor suppressor genes. **a** Mutation pairs that have impacts on RFS. Color bars indicate normalized hazard ratio. **b** Mutation pairs that have impacts on OS. Color bars indicate normalized hazard ratio. **c** Number of genes that have significant impacts on RFS when co-mutating with specific tumor suppressor genes. **d** Number of genes that have significant impacts on OS when co-mutating with specific tumor suppressor genes. OS, overall survival; RFS, recurrence-free survival; Statistical significance was assessed using *Log-rank test*, * $P < 0.05$, ** $P < 0.01$, *** $P < 0.001$.
