# Supplementary material for: Genomic and transcriptomic dynamics in the stepwise progression of lung adenocarcinoma
Source: Cell Res. 2025 Dec 4;35(12):1037–55. doi: 10.1038/s41422-025-01200-w (PMC12689645; doi:10.1038/s41422-025-01200-w)
Supplement: Supplementary file 12 — Supplementary information, Fig. S12 [file 41422_2025_1200_MOESM12_ESM.pdf]

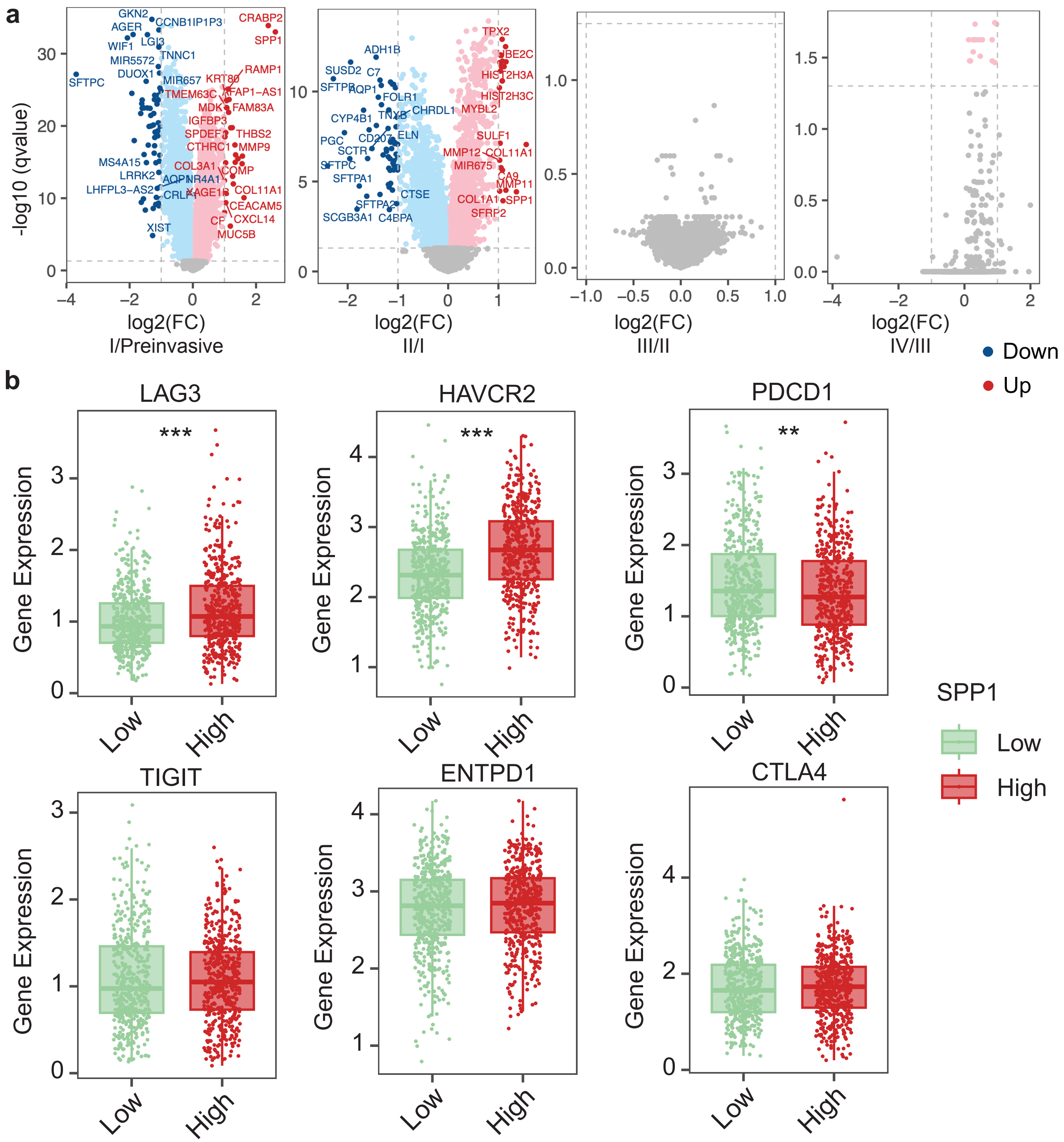

Fig. S12 Stepwise transcriptomic changes in the progression of lung adenocarcinoma. a Differentially expressed genes between each two adjacent pathological stages. From left to right: differentially expressed genes between stage I and pre-invasive samples; between stage II and stage I samples; between stage III and stage II samples; and between stage IV and stage III samples. **b** Expression of LAG3, HAVCR2, PDCD1, TIGIT, ENTPD1 and CTLA4 between samples with high and low expression of SPP1. Statistical significance was assessed using *Wilcoxon test*. ** $P < 0.01$, *** $P < 0.001$.
