# Supplementary figures and images for: Genomic and transcriptomic dynamics in the stepwise progression of lung adenocarcinoma
Source: Cell Res. 2025 Dec 4;35(12):1037–55. doi: 10.1038/s41422-025-01200-w (PMC12689645; doi:10.1038/s41422-025-01200-w)

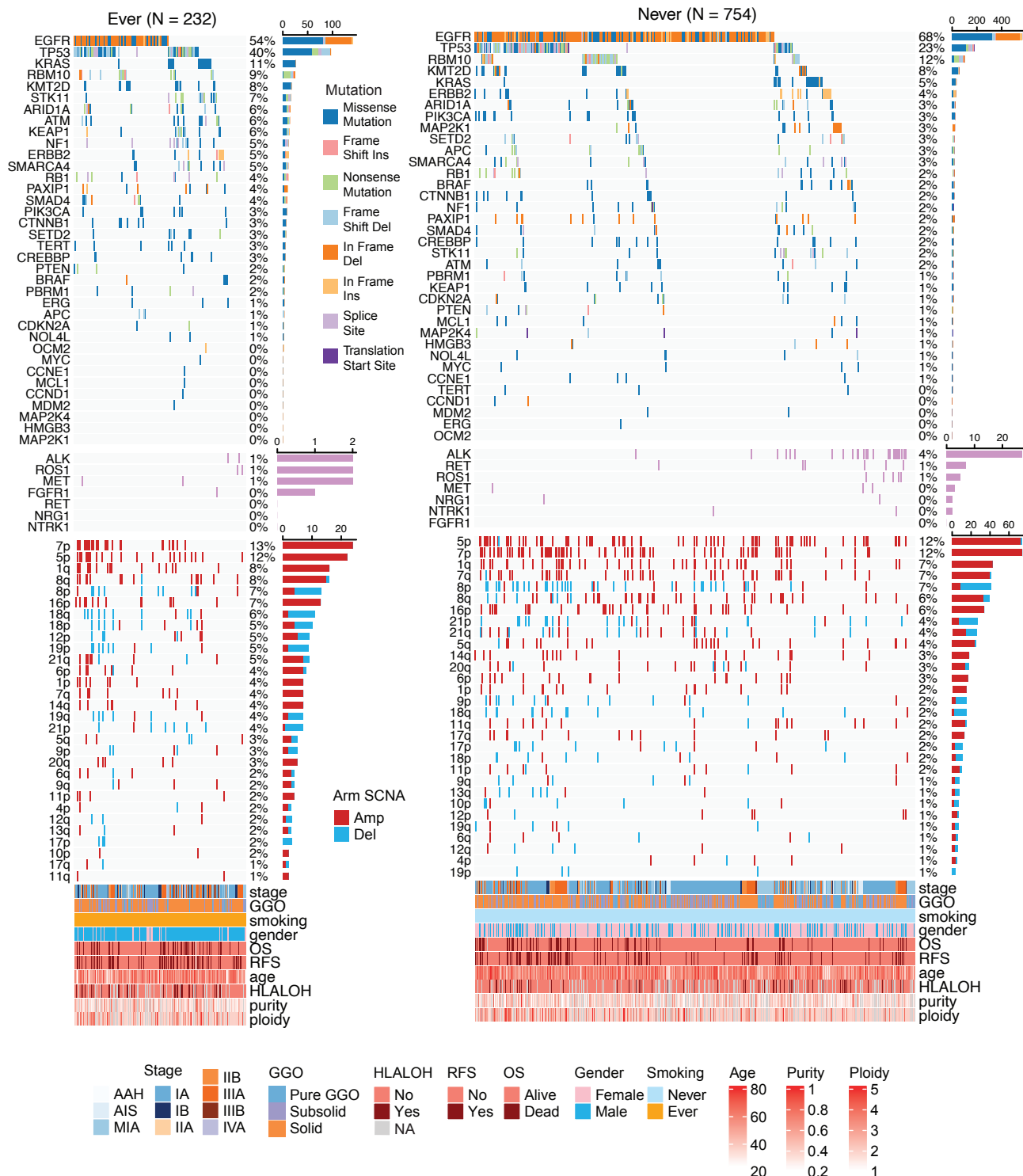

**Fig. S13** Mutational landscape of ever-smokers (left) and never-smokers (right).

Supplement: Supplementary file 13 — Supplementary information, Fig. S13 [file 41422_2025_1200_MOESM13_ESM.pdf]
