# Supplementary material for: Genomic and transcriptomic dynamics in the stepwise progression of lung adenocarcinoma
Source: Cell Res. 2025 Dec 4;35(12):1037–55. doi: 10.1038/s41422-025-01200-w (PMC12689645; doi:10.1038/s41422-025-01200-w)
Supplement: Supplementary file 14 — Supplementary information, Fig. S14 [file 41422_2025_1200_MOESM14_ESM.pdf]

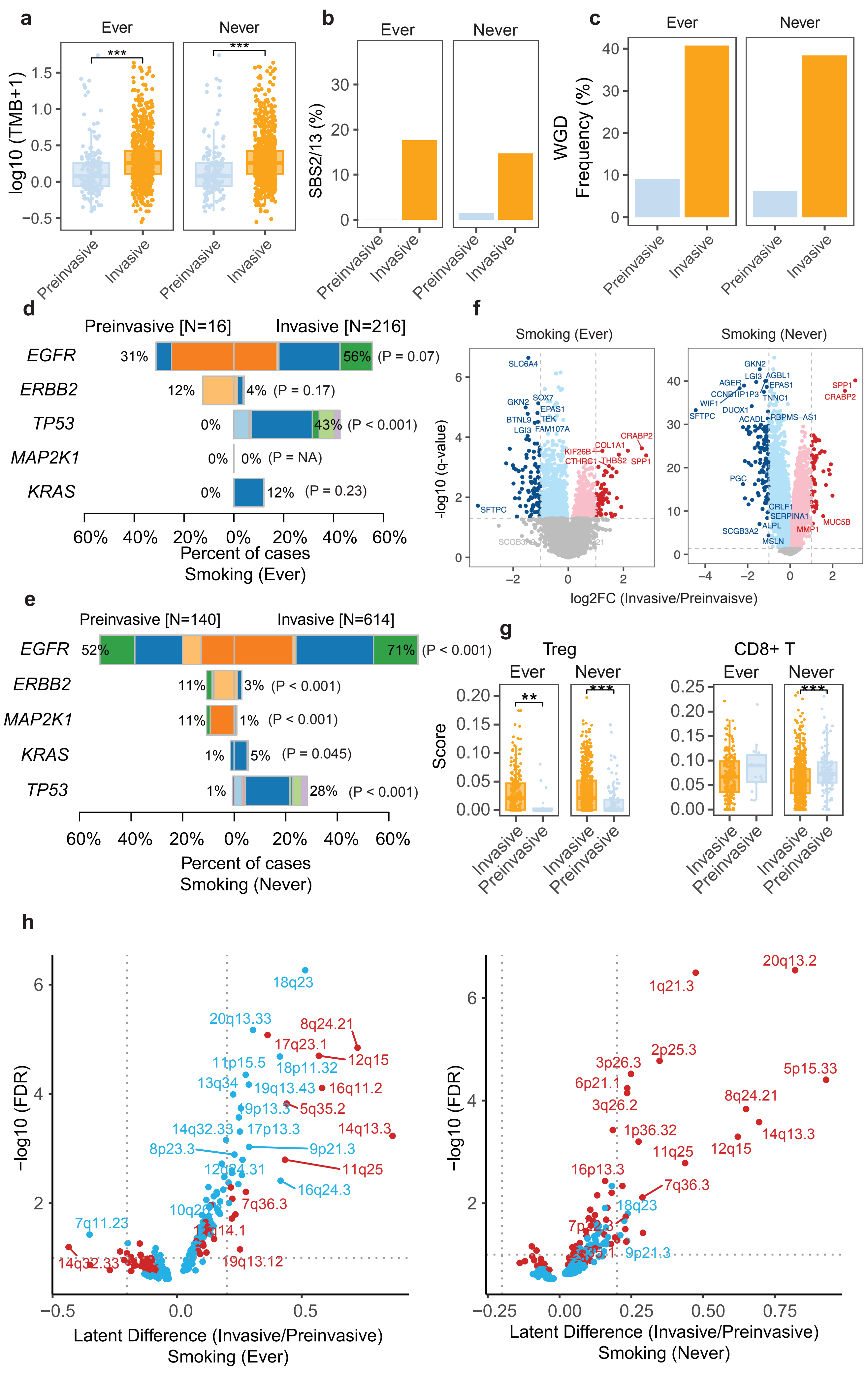

Fig. S14 Comparison of genomic and transcriptomic changes between pre-invasive and invasive samples, grouped by smoking status. a Comparison of tumor mutation burden (TMB) between pre-invasive and invasive samples in ever-smokers (left) and never-smokers (right). **b** Comparison of APOBEC signature (SBS2/13) activities between pre-invasive and invasive samples in ever-smokers (left) and never-smokers (right). **c** Comparison of whole genome doubling (WGD) events between pre-invasive and invasive samples in ever-smokers (left) and never-smokers (right). **d** Comparison of mutation frequencies of major driver genes between pre-invasive and invasive samples in ever-smokers. **e** Comparison of mutation frequencies of major driver genes between pre-invasive and invasive samples in never-smokers. **f** Differentially expressed genes between invasive and pre-invasive adenocarcinomas in ever-smokers (left) and never-smokers (right). **g** Comparison of Treg (left) and CD8+ T (right) signatures between invasive and pre-invasive adenocarcinomas in ever-smokers and never-smokers. **h** Comparison of somatic copy number alteration events between pre-invasive and invasive adenocarcinoma in ever-smokers (left) and never-smokers (right). Statistical significance was assessed using Wilcoxon test, ** $P < 0.01$, *** $P < 0.001$.
