# Supplementary material for: Genomic and transcriptomic dynamics in the stepwise progression of lung adenocarcinoma
Source: Cell Res. 2025 Dec 4;35(12):1037–55. doi: 10.1038/s41422-025-01200-w (PMC12689645; doi:10.1038/s41422-025-01200-w)
Supplement: Supplementary file 15 — Supplementary information, Fig. S15 [file 41422_2025_1200_MOESM15_ESM.pdf]

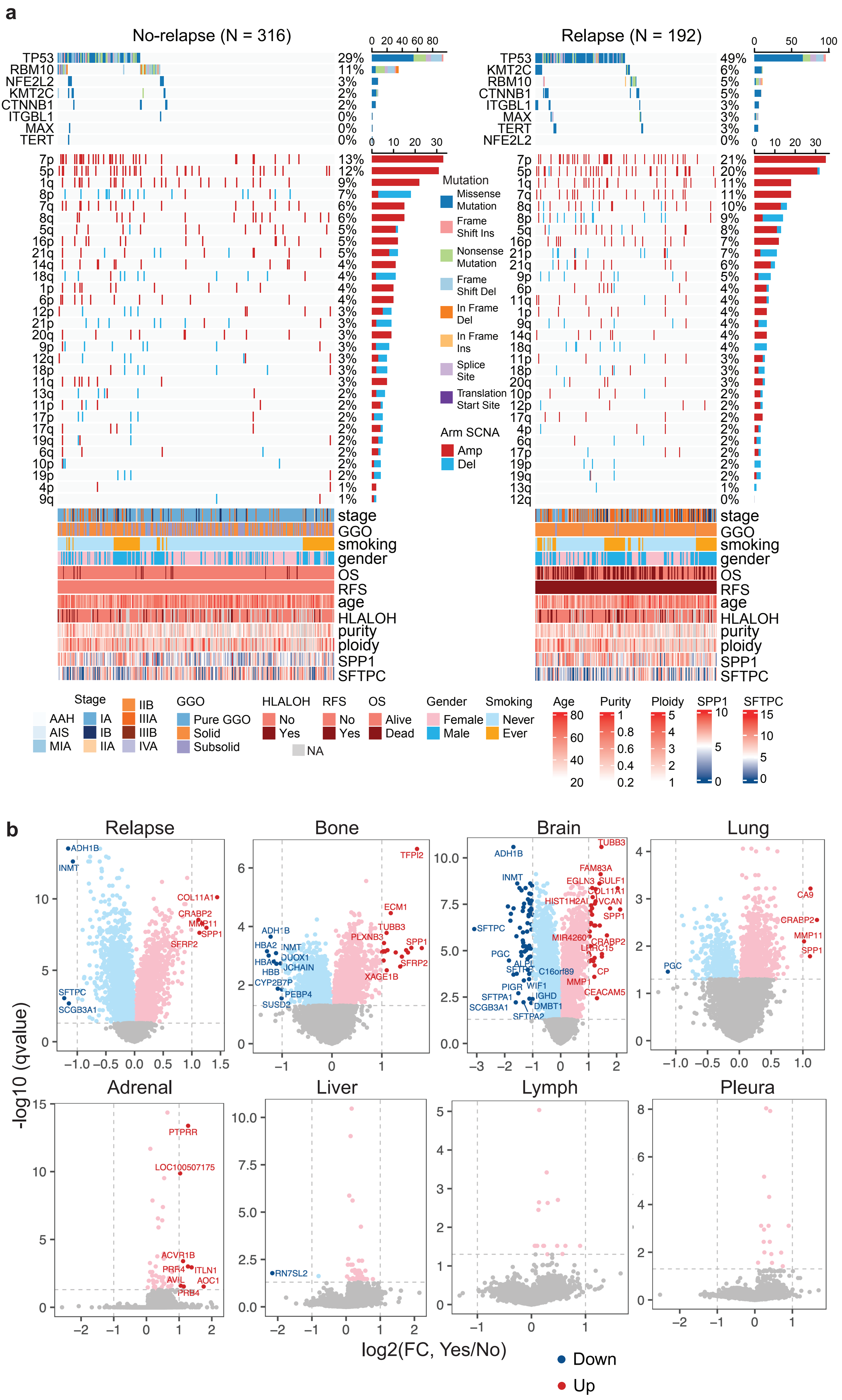

Fig. S15 Comparison of genomic and transcriptomic changes between patients with metastasis and those without metastasis in at least 5 years follow-up time. **a** Landscape of somatic mutations and SCNAs in patients with metastasis and those without metastasis in at least 5 years follow-up time. **b** Differentially expressed genes between patients with metastasis and those without metastasis in at least 5 years follow-up time. The first panel compares patients with vs without metastasis, while the other panels compare patients with site-specific metastasis vs without metastasis.
