# Supplementary material for: Genomic and transcriptomic dynamics in the stepwise progression of lung adenocarcinoma
Source: Cell Res. 2025 Dec 4;35(12):1037–55. doi: 10.1038/s41422-025-01200-w (PMC12689645; doi:10.1038/s41422-025-01200-w)
Supplement: Supplementary file 16 — Supplementary information, Fig. S16 [file 41422_2025_1200_MOESM16_ESM.pdf]

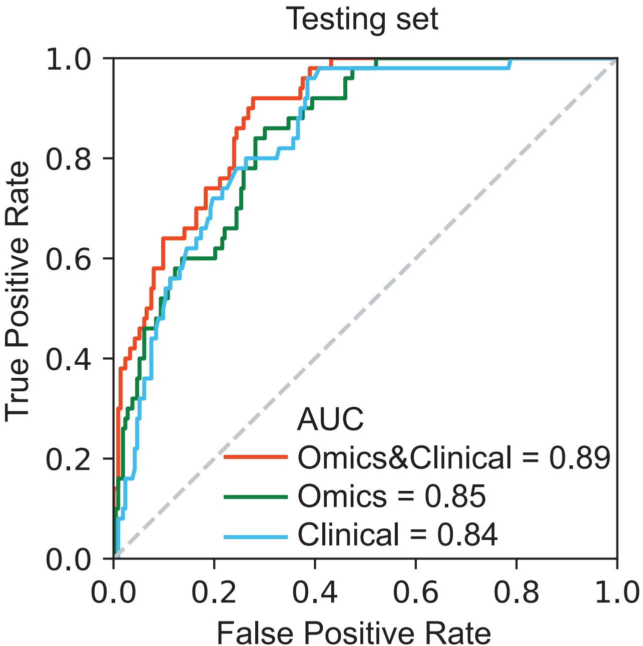

Fig. S16 ROC curves for predicting LUAD relapse based on omics data, clinical variables, and merged data, in the testing set.
