# Supplementary material for: Genomic and transcriptomic dynamics in the stepwise progression of lung adenocarcinoma
Source: Cell Res. 2025 Dec 4;35(12):1037–55. doi: 10.1038/s41422-025-01200-w (PMC12689645; doi:10.1038/s41422-025-01200-w)
Supplement: Supplementary file 17 — Supplementary information, Fig. S17 [file 41422_2025_1200_MOESM17_ESM.pdf]

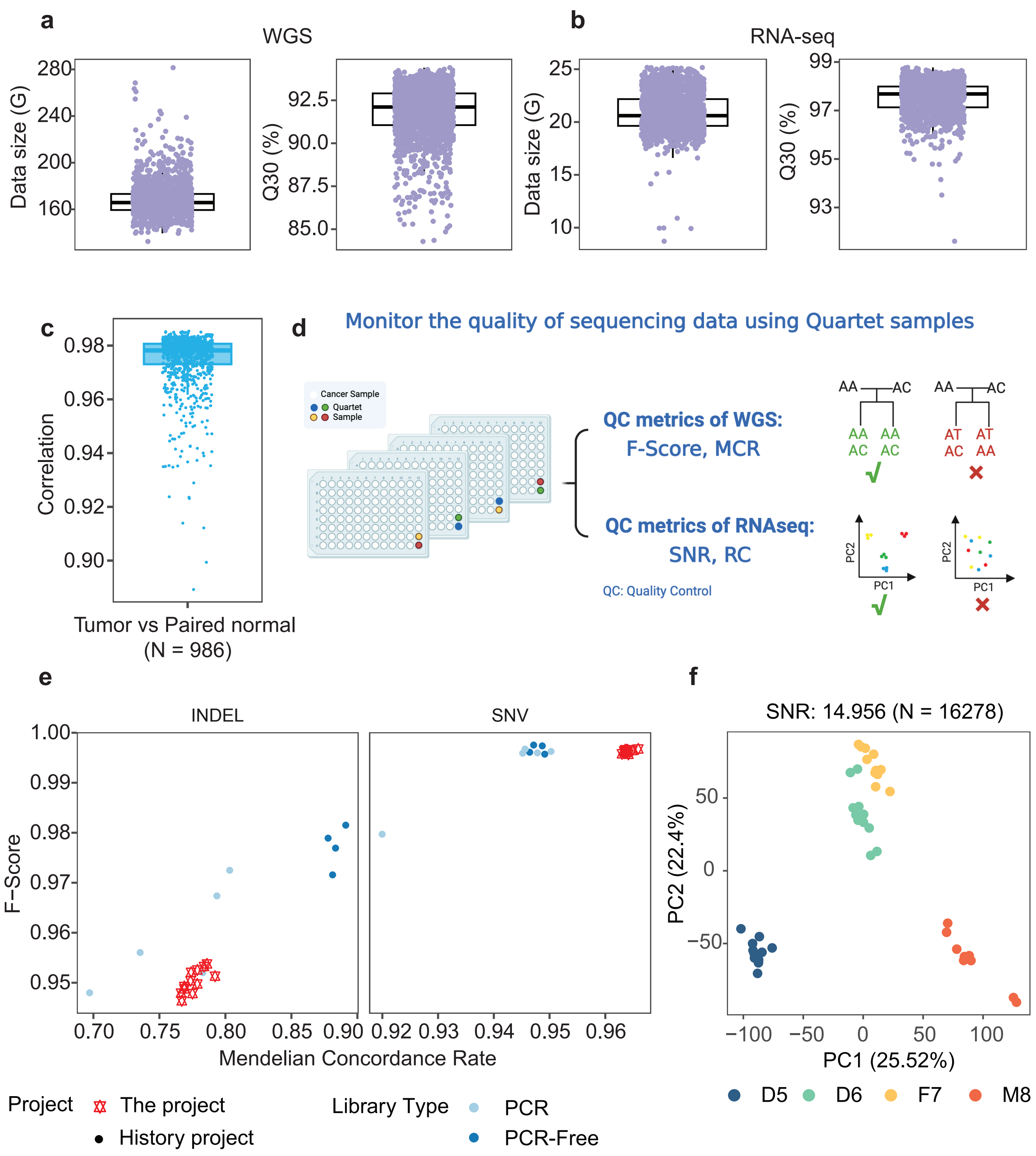

Fig. S17 WGS and RNA-seq data quality control. a Data size and Q30 scores for all WGS samples. **b** Data size and Q30 scores for all RNA-seq samples. **c** Pairwise detection of tumor and normal samples in WGS data. **d** Quality control processes during data generation, monitored using Quartet's DNA and RNA Reference Standard Material. **e** Distribution of F-score and Mendelian Concordance Rate for WGS data, evaluated using Quartet DNA standard material throughout the entire WGS data generation process for tumor samples. **f** PCA and SNR results for four sample types in Quartet RNA Reference Standard Material RNA-seq data, encompassing the full RNA-seq data generation process for tumor samples.
