# Supplementary material for: Genomic and transcriptomic dynamics in the stepwise progression of lung adenocarcinoma
Source: Cell Res. 2025 Dec 4;35(12):1037–55. doi: 10.1038/s41422-025-01200-w (PMC12689645; doi:10.1038/s41422-025-01200-w)
Supplement: Supplementary file 18 — Supplementary information, Fig. S18 [file 41422_2025_1200_MOESM18_ESM.pdf]

a

	None (N=307)	Amp (N=307)	P-value
**purity**			
Mean (SD)	0.403 (0.135)	0.407 (0.142)	0.734
Median [Min, Max]	0.380 [0.200, 0.990]	0.380 [0.200, 0.850]	
**RIN**			
Mean (SD)	5.49 (1.61)	5.54 (1.69)	0.76
Median [Min, Max]	5.80 [1.00, 9.10]	5.80 [1.00, 9.40]	

	Preinvasive (N=110)	Invasive (N=110)	P-value
**purity**			
Mean (SD)	0.351 (0.128)	0.353 (0.127)	0.907
Median [Min, Max]	0.330 [0.210, 0.990]	0.330 [0.200, 0.990]	
**RIN**			
Mean (SD)	5.72 (1.40)	5.82 (1.36)	0.591
Median [Min, Max]	5.80 [1.00, 8.50]	5.95 [1.00, 9.00]	

	GGO (N=323)	Solid (N=323)	P-value
**purity**			
Mean (SD)	0.393 (0.150)	0.393 (0.142)	0.972
Median [Min, Max]	0.360 [0.200, 0.990]	0.360 [0.200, 0.980]	
**RIN**			
Mean (SD)	5.42 (1.78)	5.42 (1.68)	0.993
Median [Min, Max]	5.70 [1.00, 9.60]	5.60 [1.00, 9.30]	

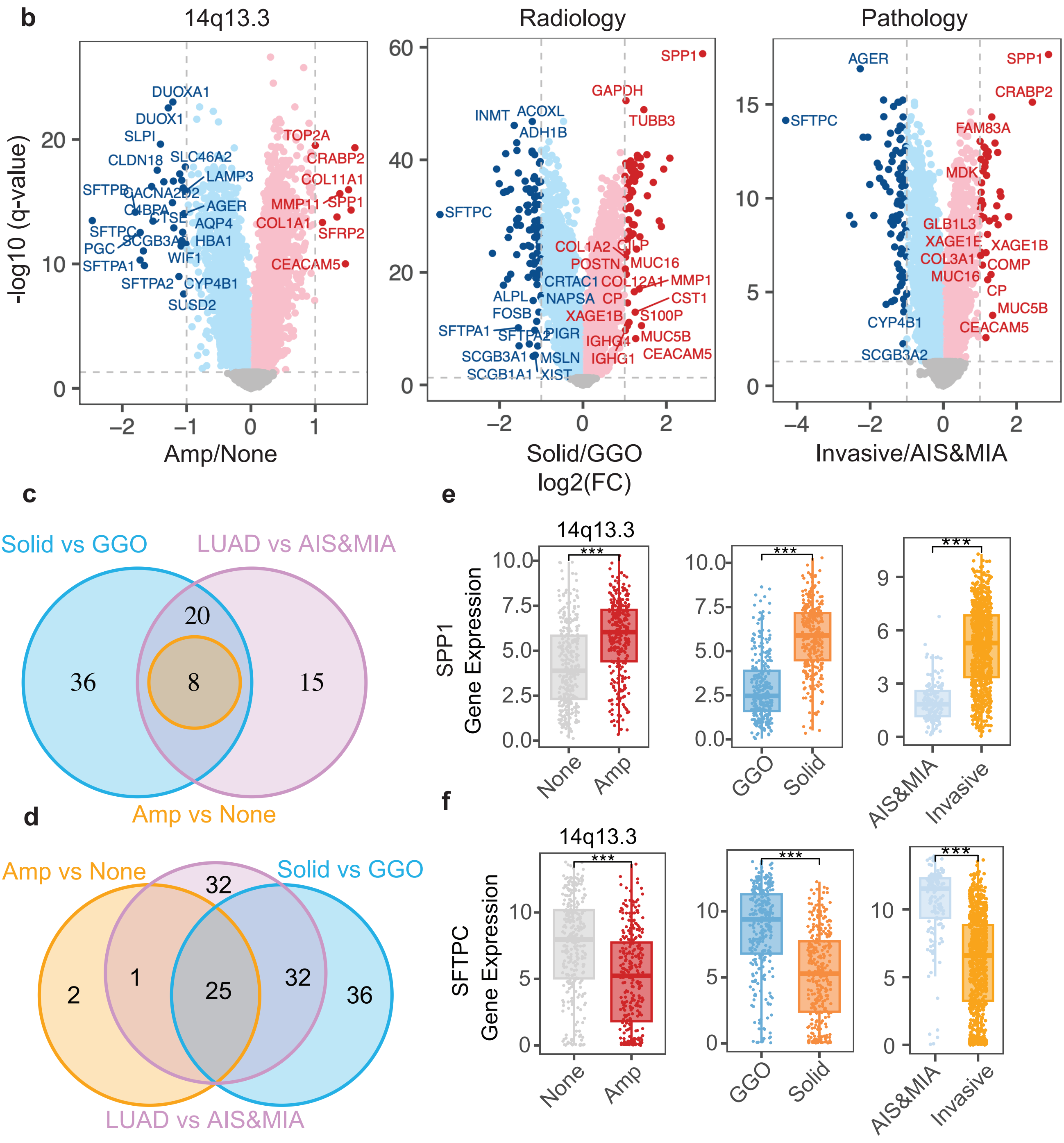

Fig. S18 Key transcriptomic changes that were associated with LUAD progression after adjusting for RIN and tumor purity. a The clinical information table demonstrates no significant differences in RIN values and tumor purity among the three comparison groups: pre-invasive versus invasive, GGO versus solid, and 14q13.3 copy number amplification versus non-amplification groups. **b** Differential expressed genes between samples having chromosome 14q13.3 amplifications or not (left), samples manifesting as solid nodules vs GGOs (middle), and invasive vs pre-invasive samples (right). **c** Venn diagram demonstrating significantly up-regulated genes in all the 3 comparisons. **d** Venn diagram demonstrating significantly down-regulated genes in all the 3 comparisons. **e** Comparison of SPP1 expression in samples having chromosome 14q13.3 amplifications or not (left), samples manifesting as solid nodules vs GGOs (middle), and invasive vs pre-invasive samples (right). **f** Comparison of SFTPC expression in samples having chromosome 14q13.3 amplifications or not (left), samples manifesting as solid nodules vs GGOs (middle), and invasive vs pre-invasive samples (right). *** $P < 0.001$.
