# Supplementary material for: Genomic and transcriptomic dynamics in the stepwise progression of lung adenocarcinoma
Source: Cell Res. 2025 Dec 4;35(12):1037–55. doi: 10.1038/s41422-025-01200-w (PMC12689645; doi:10.1038/s41422-025-01200-w)
Supplement: Supplementary file 1 — Supplementary information, Fig. S1 [file 41422_2025_1200_MOESM1_ESM.pdf]

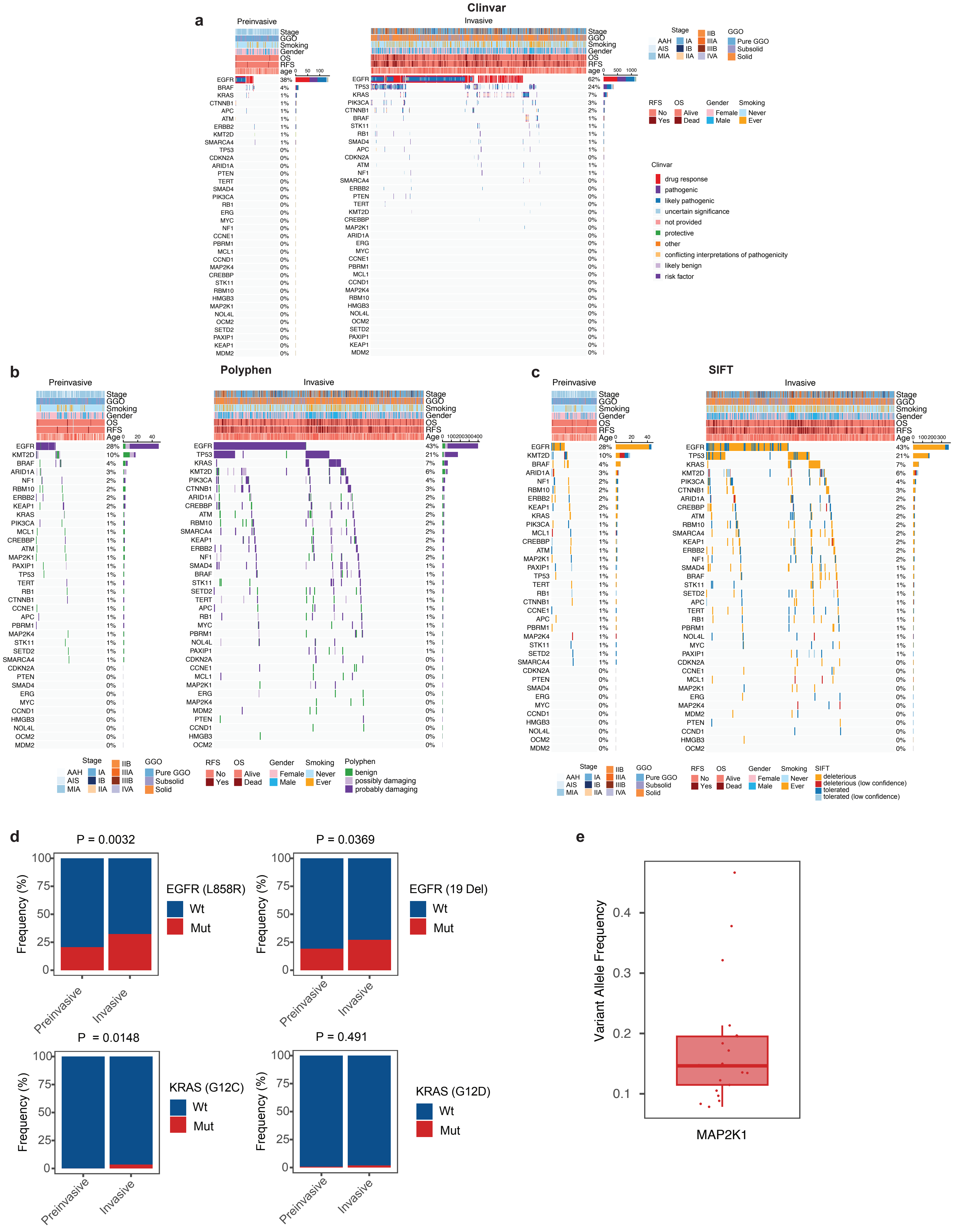

Fig. S1. Somatic mutation landscape and frequency analysis in preinvasive and invasive lung adenocarcinoma. a-c Mutations are categorized based on their clinical significance according to ClinVar (a), PolyPhen (b), and SIFT (c). **d** Comparison of mutation frequencies in EGFR L858R, *EGFR Exon 19 deletion*, *KRAS G12C* and *KRAS G12D* between pre-invasive and invasive adenocarcinomas. **e** Boxplot showing the distribution of variant allele frequencies (VAF) for mutations detected in the *MAP2K1* gene. Statistical significance was assessed using *Fisher's exact test*.
