# Supplementary material for: Genomic and transcriptomic dynamics in the stepwise progression of lung adenocarcinoma
Source: Cell Res. 2025 Dec 4;35(12):1037–55. doi: 10.1038/s41422-025-01200-w (PMC12689645; doi:10.1038/s41422-025-01200-w)
Supplement: Supplementary file 6 — Supplementary information, Fig. S6 [file 41422_2025_1200_MOESM6_ESM.pdf]

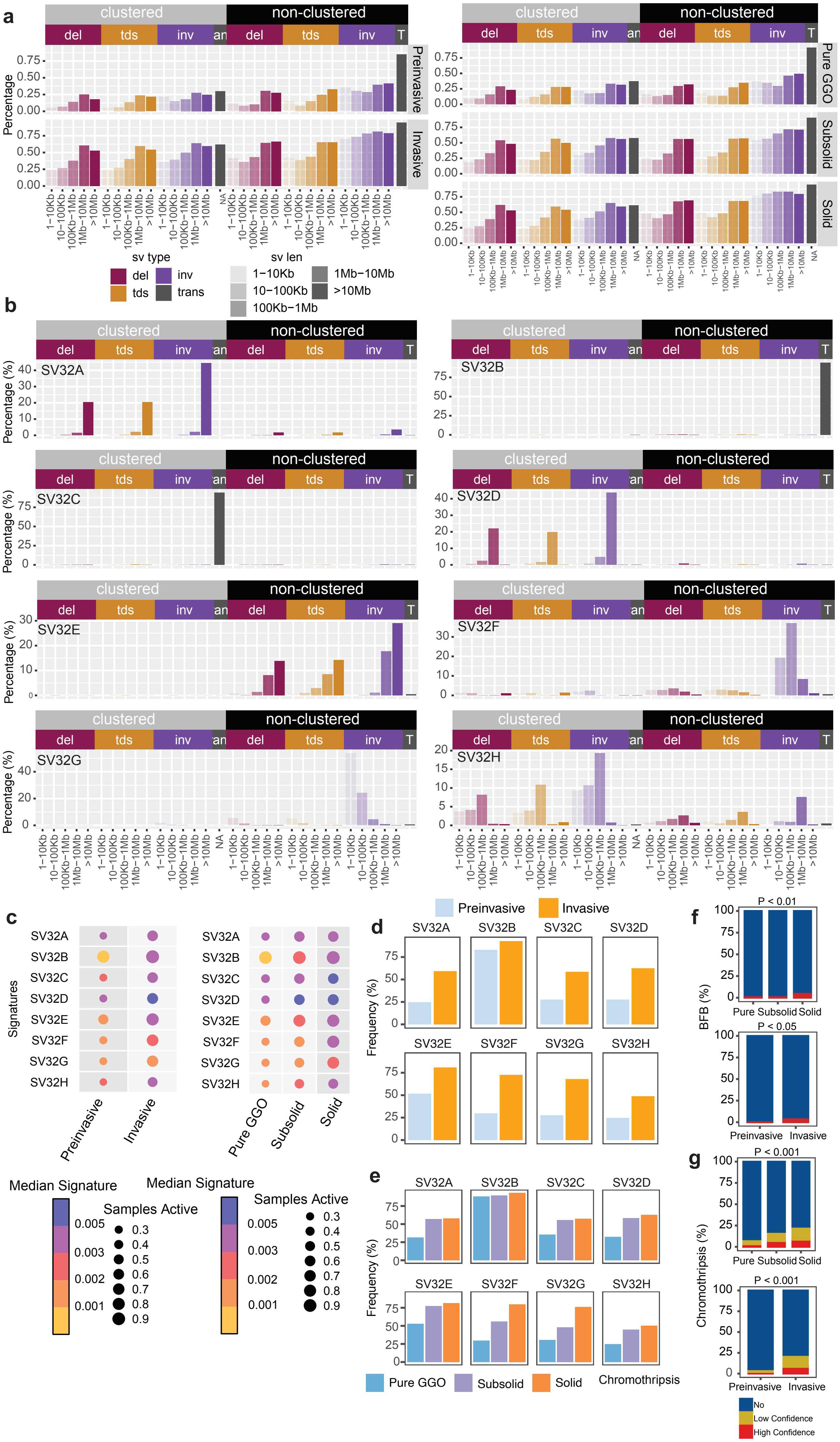

Fig. S6 Characteristics of structural variations (SVs) across different pathological and radiological stages. a Frequency distribution of different lengths and SV types across pathological and radiological stages. **b** Eight SV signature characteristics. **c** Load and frequency of eight SV signature characteristics across pathological and radiological stages. **d-e** Frequency distribution of eight SV signature characteristics across pathological stages (d) and radiological stages (e). **f-g** Frequency distribution of breakage-fusion-bridge (BFB) structural variants (f), and chromothripsis events (g) across pathological and radiological stages.
